# Plk1 Self-Organization and Priming Phosphorylation of HsCYK-4 at the Spindle Midzone Regulate the Onset of Division in Human Cells

**DOI:** 10.1371/journal.pbio.1000111

**Published:** 2009-05-26

**Authors:** Mark E. Burkard, John Maciejowski, Verónica Rodriguez-Bravo, Michael Repka, Drew M. Lowery, Karl R. Clauser, Chao Zhang, Kevan M. Shokat, Steven A. Carr, Michael B. Yaffe, Prasad V. Jallepalli

**Affiliations:** 1 Molecular Biology Program, Memorial Sloan-Kettering Cancer Center, New York, New York, United States of America; 2 Department of Medicine, Memorial Sloan-Kettering Cancer Center, New York, New York, United States of America; 3 Departments of Biology and Biological Engineering, Center for Cancer Research, Massachusetts Institute of Technology, Cambridge, Massachusetts, United States of America; 4 Broad Institute of MIT and Harvard, Cambridge, Massachusetts, United States of America; 5 Howard Hughes Medical Institute, University of California at San Francisco, San Francisco, California, United States of America; 6 Department of Cellular and Molecular Pharmacology, University of California at San Francisco, San Francisco, California, United States of America; Dana-Farber Cancer Institute, United States of America

## Abstract

Animal cells initiate cytokinesis in parallel with anaphase onset, when an actomyosin ring assembles and constricts through localized activation of the small GTPase RhoA, giving rise to a cleavage furrow. Furrow formation relies on positional cues provided by anaphase spindle microtubules (MTs), but how such cues are generated remains unclear. Using chemical genetics to achieve both temporal and spatial control, we show that the self-organized delivery of Polo-like kinase 1 (Plk1) to the midzone and its local phosphorylation of a MT-bound substrate are critical for generating this furrow-inducing signal. When Plk1 was active but unable to target itself to this equatorial landmark, both cortical RhoA recruitment and furrow induction failed to occur, thus recapitulating the effects of anaphase-specific Plk1 inhibition. Using tandem mass spectrometry and phosphospecific antibodies, we found that Plk1 binds and directly phosphorylates the HsCYK-4 subunit of centralspindlin (also known as MgcRacGAP) at the midzone. At serine 157, this modification creates a major docking site for the tandem BRCT repeats of the Rho GTP exchange factor Ect2. Cells expressing only a nonphosphorylatable form of HsCYK-4 failed to localize Ect2 at the midzone and were severely impaired in cleavage furrow formation, implying that HsCYK-4 is Plk1's rate-limiting target upstream of RhoA. Conversely, tethering an inhibitor-resistant allele of Plk1 to HsCYK-4 allowed furrows to form despite global inhibition of all other Plk1 molecules in the cell. Our findings illuminate two key mechanisms governing the initiation of cytokinesis in human cells and illustrate the power of chemical genetics to probe such regulation both in time and space.

## Abstract 2

Self-regulated movement of Polo-like kinase 1 to the midzone of the mitotic spindle initiates a local signaling cascade that activates the cell division machinery at the cell's equator.

## Author Summary

During mitosis, the separation of duplicated chromosomes and subsequent cytokinesis (cell division) are tightly coupled processes. Cytokinesis must occur not only after chromosomes have separated but also in the physical space between the chromosomes, so that each daughter cell inherits the appropriate genetic material. The mechanisms responsible for this cellular choreography are poorly understood, however. We used chemical genetics to dissect the role of a key regulator of cell division, Polo-like kinase 1 (Plk1) in human cells. We show that, contrary to previous models, the ability of Plk1 to seek out microtubules that lie between the separated chromosomes (so-called midzone microtubules) provides the cell with an affirmative command to divide. Once assembled at this landmark, Plk1 phosphorylates HsCYK-4, a component of the centralspindlin complex (so named because it assembles at the spindle midzone) and enables binding between HsCYK-4 and Ect2, another regulator of cell division. Bound Ect2 then communicates with the machinery that assembles the actin- and myosin-based contractile ring, leading to division of the cell into two daughters. Our work therefore reveals new insights into how Plk1 temporally and spatially orchestrates division of human cells.

## Introduction

At the end of mitosis, a physical barrier must be established between the segregated sister genomes to produce two distinct cells. This process, termed cytokinesis, involves the assembly and constriction of an actomyosin ring at the cell equator [1]–[3]. In addition to its fundamental role in cell multiplication, cytokinesis contributes to genome integrity, because cells that fail to complete cytokinesis often reduplicate their chromosomes and centrosomes in the following cell cycle. Such tetraploid cells are prone to further genomic instability and display increased propensity for oncogenic transformation in vitro and in vivo [4],[5].

In metazoans, cytokinesis is intimately linked to the structure of the anaphase spindle [6]. Both astral and spindle midzone microtubules (MTs) provide cues that direct contractile ring assembly to the equator, although the relative contribution of these two MT populations has been hotly debated over the past 40 years [7],[8]. Recent evidence indicates that both mechanisms can be active simultaneously and contribute redundantly to furrow positioning [9]–[11].

In addition to positional cues provided by the anaphase spindle, cytokinesis requires regulated changes in protein phosphorylation. For example, declining Cdk1 activity at anaphase onset allows the reversal of inhibitory phosphorylations on PRC1 and the MKLP1 subunit of centralspindlin, enabling these factors to bind and bundle antiparallel MTs that constitute the spindle midzone [12],[13]. The other subunit of centralspindlin, known as HsCYK-4 or MgcRacGAP, contains a GTPase activator protein (GAP) domain and directly interacts with the GTP exchange factor (GEF) Ect2, and both HsCYK-4 and Ect2 are essential for equatorial recruitment of the RhoA GTPase, the master regulator of actomyosin dynamics during cytokinesis [14]–[19].

In contrast to Cdk1, other mitotic kinases, including Polo-like kinase 1 (Plk1) and Aurora B, regulate cytokinesis positively [1]–[3],[20]–[22]. Because chronic Plk1 inactivation results in profound mitotic defects that trigger the spindle checkpoint and cause a terminal cell-cycle arrest in prometaphase, Plk1's role during the initiation of cytokinesis was not discovered until the recent advent of fast-acting chemical inhibitors [23]–[27]. When applied to cells at the metaphase-to-anaphase transition, these compounds rapidly suppress Plk1 activity, cause mislocalization of Ect2, and block RhoA-dependent initiation of contractile ring assembly [24],[27]–[29]. However, the substrates and biochemical mechanisms that account for these inhibitor-induced phenotypes remain unknown.

A second and related issue concerns the spatial regulation of Plk1 itself. While Plk1 predominantly resides at kinetochores and centrosomes in prometaphase cells, it rapidly redistributes onto the spindle midzone at anaphase onset [30]. This localization pattern stems from Plk1's synergistic phosphorylation of and docking with multiple midzone components via its Polo-box domain (PBD), which is a specialized phosphopeptide-binding module [31],[32]. Accordingly, inhibiting Plk1 activity in anaphase cells disrupts this positive-feedback mechanism and prevents Plk1's targeting to the midzone [24],[27]–[29]. Nevertheless, the relationship between Plk1's spatial auto-regulation and the molecular events leading to RhoA activation and contractile ring assembly remains unsettled, because the PBD is also required for Plk1's upstream functions in prometaphase, without which cells are unable to satisfy the spindle checkpoint [33],[34]. Based on studies in which Plk1's midzone localization was indirectly perturbed through the depletion of individual docking partners such as PRC1 [35]–[37] or co-expression of dominant-negative Plk1 and spindle checkpoint constructs [34], current models for cell division presume that this self-organization is not required to recruit Ect2 to the midzone or initiate contractile ring assembly [1],[37]–[39]. However, this interpretation is complicated by the fact that both dominant-negative and RNAi assays are susceptible to incomplete penetrance and thus false-negative results, especially in signaling pathways characterized by strong positive feedback and competition among multiple binding partners and substrates [40],[41]. For example, reducing Plk1 levels by 95% blocks chromosome bi-orientation effectively but still leaves cells with enough Plk1 to initiate furrowing if the spindle checkpoint is also down-regulated [42], whereas furrowing does not occur if Plk1 activity is directly and potently inhibited in anaphase using small molecules [24],[27]–[29].

To elucidate the spatial and molecular mechanisms by which Plk1 triggers RhoA recruitment and contractile ring assembly, we took advantage of a chemical genetic system for Plk1 inhibition [24]. This system uses *PLK1*-null human retinal pigment epithelial (RPE) cells complemented by a mutant Plk1 allele, whose catalytic pocket has been enlarged to accept bulky purine analogs as ATP-competitive inhibitors (Plk1^as^, for analog-sensitive) [24]. Exposure of Plk1^as^ cells to one such analog, 3-MB-PP1, abrogates all known mitotic and cytokinetic functions of the kinase, albeit on a 100-fold shorter time scale than gene deletion or RNAi. Importantly, 3-MB-PP1 is inert when applied to cells expressing wild-type Plk1, validating this compound as an allele-specific inhibitor in vivo [22]. In this Research Article, we leverage this system to modulate not only the timing of kinase signaling but also its geometry. We demonstrate that, contrary to previous models, Plk1's self-organization at the midzone in fact plays a crucial role in communicating the position of this equatorial landmark to the RhoA network. We also show that one key step in this communication involves the site-specific phosphorylation of the RhoGAP subunit of the centralspindlin complex, HsCYK-4, which creates a recognition site for a tandem BRCT repeat motif located at the N terminus of Ect2. Together these findings provide new insight into how Plk1 generates and amplifies the midzone-derived signal for cell division.

## Results

### A Chemical Genetic System for Manipulating the Geometry of Plk1 Activity in Anaphase Cells

**Figure 1:**
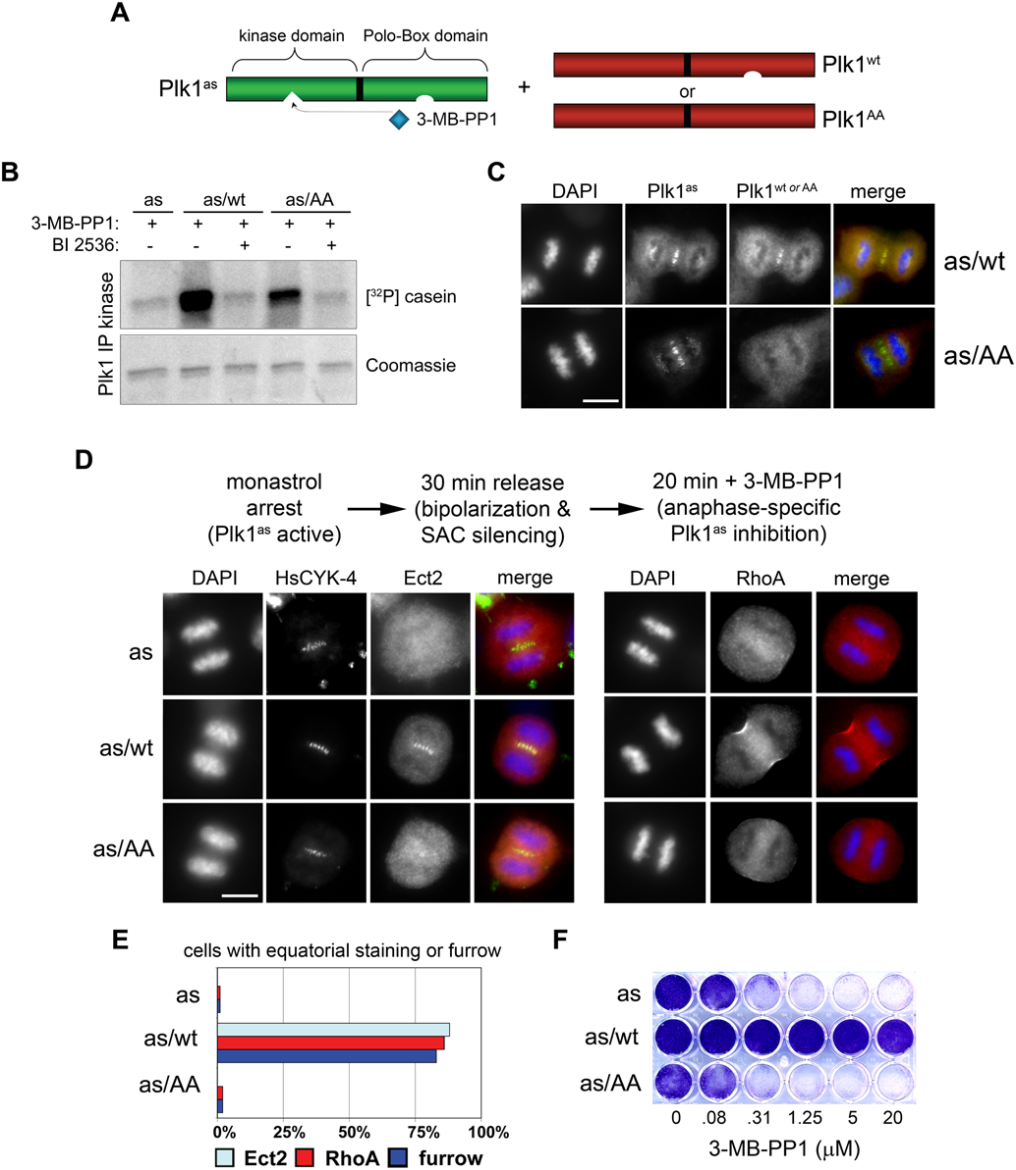
Self-assembly of Plk1 at the spindle midzone is required to activate the Ect2-RhoA network and induce formation of cleavage furrows. (A) Human *PLK1* ^Δ/Δ^ cells were reconstituted with an EGFP-tagged analog-sensitive Plk1 allele (Plk1^as^) and mCherry-tagged wild-type (Plk1^wt^) or pincer-defective (Plk1^AA^) alleles that are analog-resistant. (B) Plk1 immunoprecipitates were incubated with [γ-^32^P]ATP and casein in the presence of 3-MB-PP1 with or without BI 2536. (C) Plk1^AA^ cannot associate with the spindle midzone. Plk1^as/wt^ and Plk1^as/AA^ cells were stained with EGFP- and mCherry-specific antibodies and processed for immunofluorescence as outlined in the Materials and Methods section. Scale bar, 10 µm. (D) A chemical genetic assay for cytokinesis initiation. Plk1^as^, Plk1^as/wt^, and Plk1^as/AA^ cells were synchronously released from a monastrol block and treated with 3-MB-PP1 to acutely inhibit Plk1^as^ activity in early anaphase. Midzone localization of the Ect2/HsCYK-4 complex, cortical generation of active RhoA, and furrow ingression were analyzed by immunofluorescence microscopy. Scale bar, 10 µm. (E) Quantification of cytokinesis onset. Ect2 recruitment, cortical RhoA activation, and furrow ingression were determined by immunofluorescence microscopy (*n* = 100 anaphase cells per genotype). (F) Cells were cultured in the presence of various concentrations of 3-MB-PP1 for 8 d and stained with crystal violet to assess growth.

As a first step, Plk1^as^ cells [24] were transduced with retroviruses expressing either wild-type Plk1 (Plk1^wt^) or a so-called “pincer” mutant (Plk1^AA^) that can no longer bind phosphoserine or phosphothreonine residues [32] (Figure 1A). Both proteins were expressed at similar levels, comparable to the Plk1^as^ allele (Figure S1). Both Plk1^as/wt^ and Plk1^as/AA^ cells contained a kinase activity that was resistant to 3-MB-PP1 but sensitive to BI 2536, a pharmacologic inhibitor of Plk1 and related kinases [43] (Figure 1B). However, Plk1^wt^ localized to the spindle midzone in vivo, whereas Plk1^AA^ was diffusely distributed (Figure 1C). Next, Plk1^as/wt^ and Plk1^as/AA^ cells were synchronized in prometaphase with monastrol, released to allow bipolar spindle assembly, and then briefly treated with 3-MB-PP1 to inhibit Plk1^as^ activity in anaphase (Figure 1D). Plk1^wt^ fully complemented the cytokinesis defects caused by Plk1^as^ inhibition, as judged by localization of Ect2 at the spindle midzone, recruitment of RhoA to the equatorial cortex, and furrow ingression (Figure 1D, middle row, and Figure 1E). Plk1^as/wt^ cells were also completely resistant to 3-MB-PP1 in long-term growth assays (Figure 1F). In sharp contrast, Plk1^AA^ failed to rescue either cytokinesis onset (Figure 1D, bottom row, and Figure 1E) or sensitivity to 3-MB-PP1 (Figure 1F). This failure was not attributable to a *cis*-inhibitory effect of the PBD itself, as comparable results were obtained when the entire PBD was deleted (see below). These data suggest that, contrary to previous models, the initiation of cytokinesis requires both the catalytic activity of Plk1 and its self-regulated association with one or more factors upstream of RhoA, most likely at the spindle midzone.

### The RhoGAP Subunit of Centralspindlin Is a Substrate and Binding Partner of Plk1 at the Spindle Midzone

**Figure 2:**
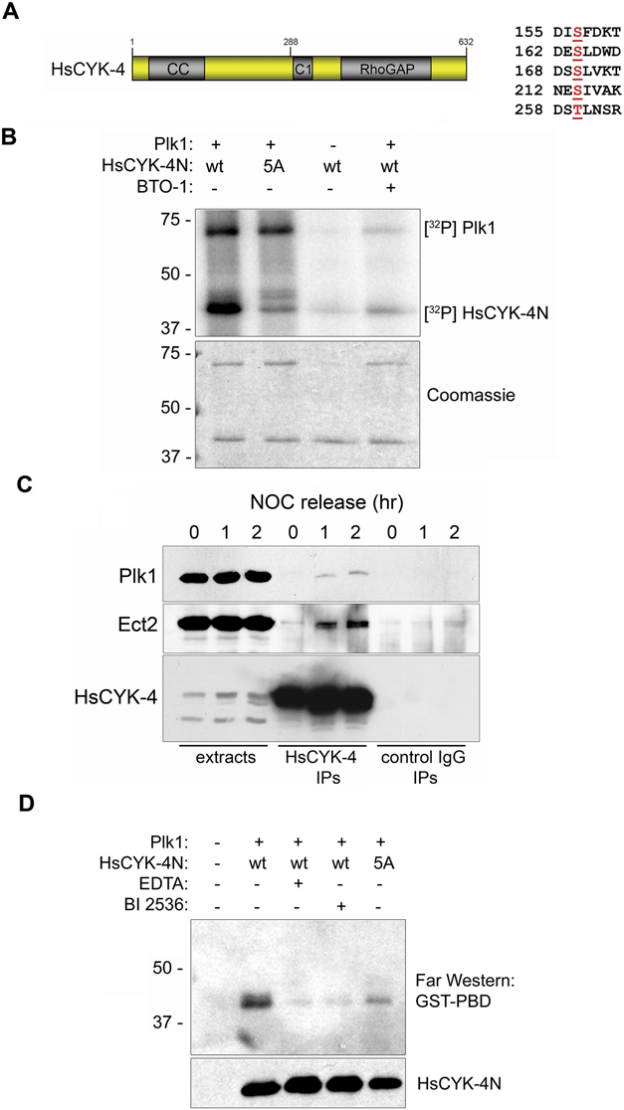
The RhoGAP subunit of the centralspindlin complex, HsCYK-4, is a substrate and binding partner of Plk1 in vitro. (A) The N terminus of HsCYK-4 contains multiple Plk1 phosphorylation sites. MS/MS spectra of Plk1-phosphorylated HsCYK-4 (1–288) are presented in Figure S2. (B) HsCYK-4 is a Plk1 substrate in vitro. Recombinant HsCYK-4 fragments were used as substrates for Plk1 kinase assays. Where indicated, BTO-1 [25] was added to inhibit Plk1 activity. (C) Plk1 associates with the Ect2/HsCYK-4 complex. HeLa cells were released from a nocodazole block and sampled at the indicated timepoints. DSP-treated whole-cell extracts were immunoprecipitated with anti-HsCYK-4 or control goat IgG and immunoblotted for Plk1 and Ect2. (D) Plk1 phosphorylation of HsCYK-4 promotes PBD docking. In vitro kinase reactions were performed as in (B), except that unlabeled ATP was used. The reaction products were resolved by SDS-PAGE and subjected to Far Western blotting using a soluble GST-PBD fusion protein as the probe. Comparable loading was verified by immunoblotting for HsCYK-4N via its chitin-binding domain (CBD) tag.

In surveying known RhoA regulators, we noted that the N terminus of HsCYK-4 contains several potential Plk1 phosphorylation sites (D/E/N-X-S/T-ϕ, where X represents any amino acid and ϕ denotes a hydrophobic residue; MBY, unpublished data, and Figure 2A) and is an excellent substrate for Plk1 in vitro (Figure 2B). These findings were of interest, because we recently isolated HsCYK-4 in an affinity-capture screen for PBD-binding proteins [44] and observed modest but reproducible coprecipitation of endogenous Plk1 and HsCYK-4 during late mitosis (Figure 2C). Tandem mass spectrometry analysis of a purified HsCYK-4 fragment (HsCYK-4N) phosphorylated by Plk1 in vitro identified four major sites (S157, S170, S214, and S260; Figure S2) that overlapped with in vivo phosphorylation sites (S157, S164, S170, and S214) detected in global phosphoproteomics analyses [45],[46]. Mutating these residues to alanine (HsCYK-4N^5A^) strongly inhibited Plk1-mediated phosphorylation in vitro (Figure 2B). To test whether phosphorylation of HsCYK-4 facilitates its recognition by the PBD, nonradioactive kinase reactions were performed, resolved by SDS-PAGE, and subjected to Far Western blotting using a GST-PBD fusion protein [44]. Whereas the Plk1-phosphorylated form of HsCYK-4 bound the soluble PBD fragment, this interaction was suppressed upon inhibition of Plk1 or mutation of all five phosphoacceptor sites (Figure 2D).

**Figure 3:**
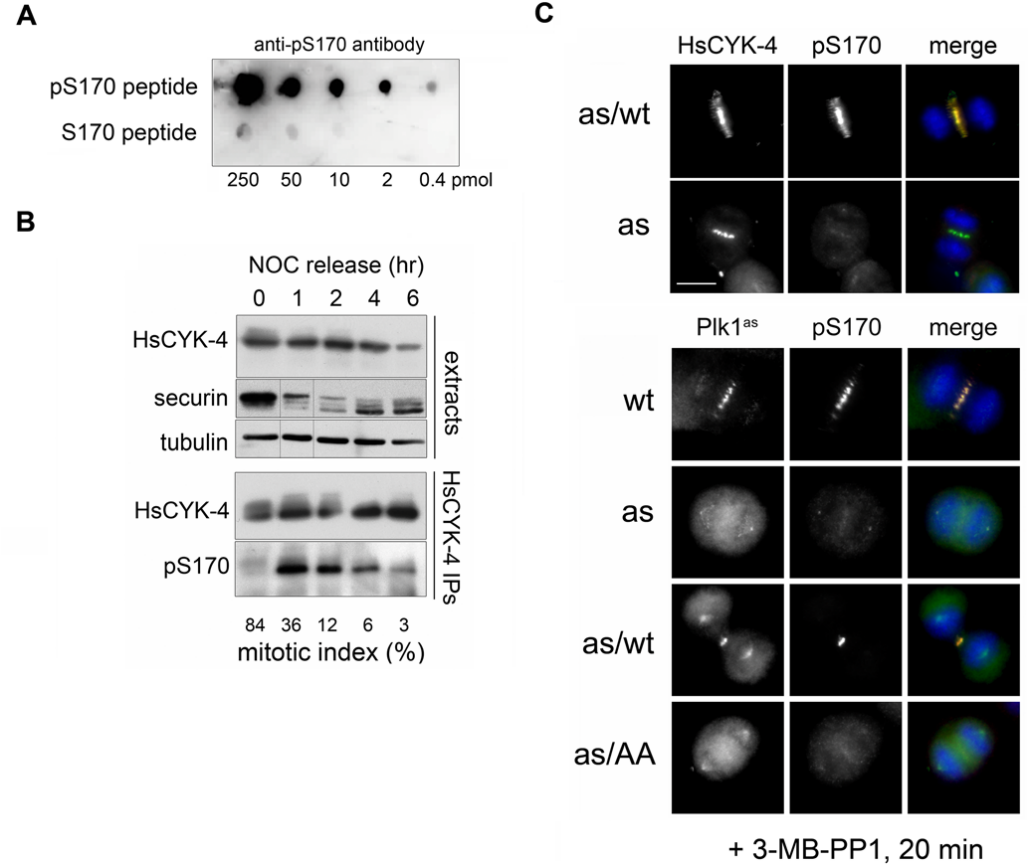
HsCYK-4 is an anaphase-specific Plk1 substrate at the midzone. (A) Validation of a pS170-specific antibody. Known amounts of pS170 or S170 (unphosphorylated) peptides were spotted on PVDF and immunoblotted with anti-pS170 antibody to assess phosphoselectivity. (B) Phosphorylation of S170 peaks during anaphase and telophase. HCT116 cells were synchronized by nocodazole block and release and either immunoblotted directly (top panels) or subjected to immunoprecipitation of total HsCYK-4, followed by immunoblot detection of pS170. Note that the securin destruction and mitotic exit data were recently reported [69] and are reproduced here to provide essential context. (C) Phosphorylation of S170 occurs on the spindle midzone and requires both the catalytic activity and self-primed association of Plk1 with the spindle midzone. RPE cells of the indicated genotypes were synchronized with monastrol, treated with 3-MB-PP1 in anaphase, and processed for immunofluorescence microscopy to visualize Plk1^as^, total HsCYK-4, and pS170. Scale bar, 10 µm.

To investigate the dynamics of HsCYK-4 phosphorylation in vivo, we raised a phosphospecific antibody against serine 170 (pS170; Figure 3A and Figure S4). Whereas this site was minimally modified in HCT116 cells that were synchronized in prometaphase with nocodazole, S170 phosphorylation increased dramatically upon release from the nocodazole arrest, as cells progressed through anaphase and cytokinesis (Figure 3B). Similar results were obtained in HeLa cells, except that higher levels of pS170 were detected during nocodazole arrest, presumably indicating a higher steady-state ratio of kinase to phosphatase activity in this cell type (Figure S3). Immunofluorescence microscopy with the pS170 antibody revealed strong phosphorylation of HsCYK-4 at the spindle midzone (Figure 3C, top panels, and Figure S4). This phosphorylation required both Plk1's catalytic activity and a functional PBD motif, as judged by the loss of the pS170 signal in 3-MB-PP1-treated Plk1^as^ and Plk1^as/AA^ cells, versus its maintenance in identically treated Plk1^as/wt^ cells (Figure 3C, bottom panels). Together, these results indicate that HsCYK-4 is a bona fide substrate and docking partner of Plk1 at the spindle midzone.

### HsCYK-4 Phosphorylation Controls Ect2 Localization and Stimulates RhoA-Dependent Contractile Ring Assembly

**Figure 4:**
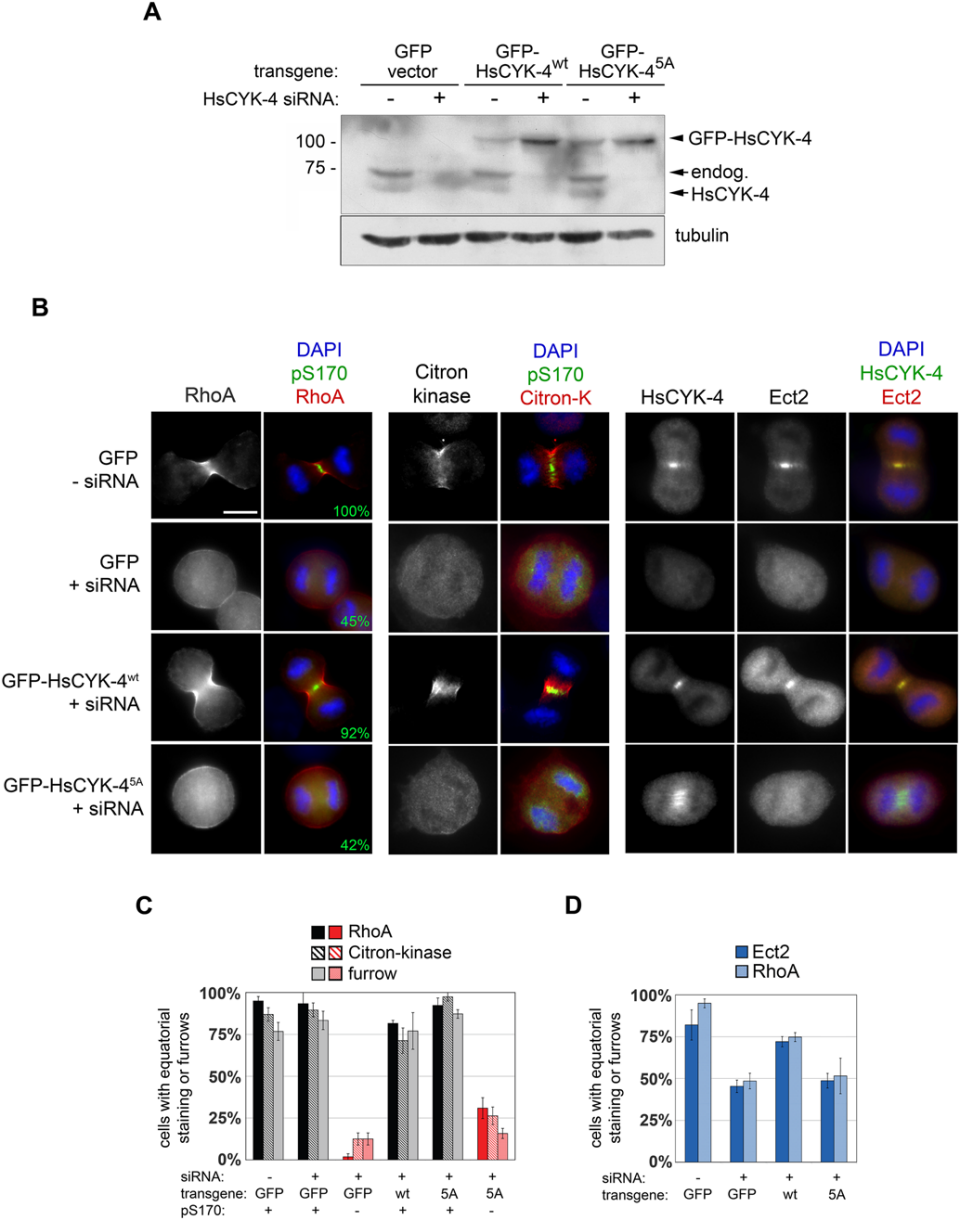
Plk1 phosphorylation of HsCYK-4 localizes Ect2 at the midzone and stimulates RhoA-dependent contractile ring assembly at the equatorial cortex. (A) Human RPE cells were stably transduced with GFP-tagged siRNA-resistant HsCYK-4 alleles, or with the empty GFP vector as a negative control, then transfected with HsCYK-4 siRNA. Samples were analyzed by SDS-PAGE and Western blotting 48 h after transfection. We observed increased levels of both GFP-tagged polypeptides after depletion of endogenous HsCYK-4, possibly due to reduced competition for MKLP1 or other stabilizing factors. Tubulin was used to confirm equal loading. (B) Cells were transfected as above, synchronized with monastrol, and fixed 60 min after release from the monastrol block. The fraction of anaphase/telophase cells with pS170 staining at the midzone is indicated (green numbers, second column) and was used to establish the efficiency of RNAi depletion and/or transgenic rescue in individual cells. The percentage of pS170-positive and -negative cells with equatorially localized proteins or ingressing furrows was quantified for each genotype. Scale bar, 10 µm. (C and D) Abrogation of HsCYK-4 phosphorylation prevents Ect2 recruitment to the spindle midzone and inhibits RhoA activation and contractile ring assembly. The percentage of anaphase/telophase cells with accumulation of Ect2 at the midzone, incorporation of active RhoA or Citron-kinase into the presumptive contractile ring, and furrow ingression was determined from four replicates in two separate experiments (*n* = 100 cells/sample); error bars denote SEM.

To determine the physiologic role of HsCYK-4 phosphorylation, human RPE cells were transduced with retroviral constructs expressing GFP-tagged and siRNA-resistant versions of HsCYK-4^wt^ or HsCYK-4^5A^. Both proteins localized to the spindle midzone, indicating their incorporation into functional centralspindlin complexes (Figure S4A). Each cell line (plus a control cell line expressing GFP alone) was transfected with HsCYK-4 siRNA and synchronized in late mitosis via timed release from a monastrol block. Although Western blotting demonstrated a profound bulk reduction in HsCYK-4 (Figure 4A), only about 50% of cells were depleted with sufficient penetrance to extinguish the pS170 phosphoepitope (Figure 4B, second column). Through careful analysis of cells co-stained for pS170 and RhoA network components, we nevertheless obtained clear evidence that the phosphorylated form of HsCYK-4 promotes the onset of cytokinesis. As expected, control cells that were effectively depleted of endogenous HsCYK-4 (and thus devoid of pS170) exhibited severe defects in concentrating RhoA at the equatorial cortex, targeting citron kinase (an established RhoA effector and myosin II activator [16],[47]) to the presumptive contractile ring, and furrow ingression (Figure 4B, second row, and Figure 4C). Expression of GFP-HsCYK-4^wt^ rescued the loss of pS170 and all other siRNA-induced phenotypes (Figure 4B, third row). In contrast, GFP-HsCYK-4^5A^ did not restore S170 phosphorylation, and these cells continued to display pronounced defects in all aspects of cytokinesis initiation (Figure 4B, bottom row, and Figure 4C). Although it was not possible to costain cells for pS170 and Ect2, both HsCYK-4-depleted and GFP-HsCYK-4^5A^ cells displayed quantitatively similar defects in recruiting Ect2 to the midzone that were proportionate to the loss of pS170 (Figure 4B and 4D). We also observed a qualitatively similar difference in the frequency of binucleated cells after siRNA transfection in asynchronous cultures (14% versus 2% for GFP-HsCYK-4^wt^; *p* = 0.001 by Fisher's exact test). Thus, Plk1's phosphorylation of HsCYK-4 plays a major role in targeting Ect2 to the spindle midzone and in stimulating RhoA-dependent contractile ring assembly at anaphase onset.

### Phosphorylation of HsCYK-4 on Serine 157 Generates a Primary Docking Site for the Tandem BRCT Repeats of Ect2

**Figure 5:**
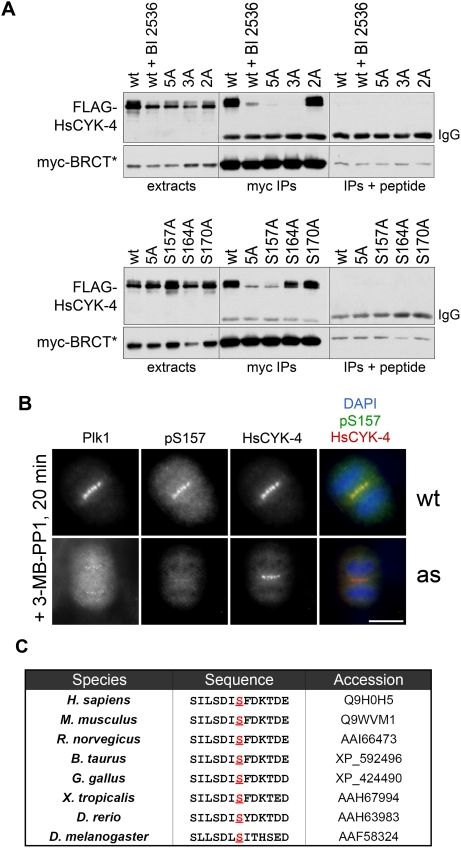
Phosphorylation of HsCYK-4 on S157 creates a docking site for the BRCT repeats on Ect2. (A) HeLa cells were cotransfected with plasmids expressing wild-type or non-phosphorylatable versions of FLAG-HsCYK-4 and a myc-tagged N-terminal fragment of Ect2 containing both BRCT repeats but lacking an inhibitory Cdk1 phosphorylation site (1-352 T342A [14]; denoted here as myc-BRCT*). Mitotic cell extracts were immunoprecipitated with anti-myc antibodies, then analyzed by SDS-PAGE and Western blotting with anti-FLAG and anti-myc antibodies to assess binding. 3A denotes the S157A/S164A/S170A triple mutant; 2A indicates the S214A/T260A double mutant. To ensure specificity, control immunoprecipitations were performed in the presence of myc peptide as a competitor. (B) Plk1 phosphorylates S157 in vivo. A pS157-specific antibody was generated (see Materials and Methods and Figure S5) and used to stain 3-MB-PP1-treated Plk1^wt^ and Plk1^as^ cells. (C) Alignment of amino acid sequences surrounding S157 in HsCYK-4 orthologs. Note that a Plk1 phosphorylation consensus site (D/E-x-S/T-ϕ) is conserved from *Drosophila* to humans.

The N terminus of Ect2 has complex regulatory functions: on the one hand, this domain is required for Ect2's binding to HsCYK-4 [14],[16] and is subject to inhibitory phosphorylation by Cdk1 [14]. On the other hand, it also tightly interacts with Ect2's C-terminal GEF domain, resulting in intramolecular auto-inhibition [48]. Structurally, this region contains two tandem BRCA1 C-terminal (BRCT) repeats, a sequence motif that, in some contexts, mediates phosphospecific protein–protein interactions [49],[50]. Thus, several distinct but nonexclusive models for how Plk1 promotes assembly of the Ect2/HsCYK-4 complex have been suggested [21],[22],[24],[29]. One possibility is that Plk1 phosphorylates Ect2 to disrupt its intramolecular association, thereby making the N-terminal domain more accessible to HsCYK-4. Another possibility is that Plk1 directly generates a phosphospecific Ect2-binding site within HsCYK-4. The mislocalization of Ect2 in HsCYK-4^5A^ mutant cells (Figure 4B) strongly supports the latter hypothesis. To test this explicitly, we cotransfected HeLa cells with epitope-tagged versions of HsCYK-4 (either wild-type or nonphosphorylatable) and an N-terminal Ect2 fragment that is immune to both intramolecular and Cdk1-dependent inhibition (myc-BRCT* [14]). Immunoprecipitation–Western blotting studies confirmed reconstitution of the complex and further showed that this interaction requires both Plk1 activity in *trans* and one major Plk1 consensus site (S157) in *cis* (Figure 5A). Similar results were also obtained using full-length Ect2 as the binding partner (Figure S6). Motivated by these findings, we generated a pS157-specific antibody and used it to confirm that this residue is indeed phosphorylated in a Plk1-dependent manner in vivo (Figure 5B and Figure S5). We conclude that Plk1 phosphorylation at S157 (an evolutionarily conserved residue in HsCYK-4 orthologs; Figure 5C) generates the primary docking site for Ect2, at least when assayed in solution. Nevertheless, other Plk1 phosphorylation sites may act in parallel with S157 to stabilize the Ect2/HsCYK-4 interaction at midzone MTs (JM and PVJ, unpublished data; M. Glotzer, personal communication).

### Plk1 Self-Organization and Phosphodependent Priming of the Ect2-Centralspindlin Complex Generate the Midzone-Based Signal for Cell Division

Together, our results suggest a simple model for how Plk1 generates the midzone-derived signal for furrow formation. At anaphase onset, Plk1 is released from its early mitotic binding partners through dephosphorylation of Cdk1-primed PBD-docking sites. Once liberated, the kinase self-organizes at the plus ends of spindle MTs by binding to and phosphorylating multiple midzone components, including the HsCYK-4 subunit of centralspindlin. Once phosphorylated on serine 157, HsCYK-4 is able to recruit Ect2 to the midzone via the latter's N-terminal BRCT repeats, thereby concentrating the RhoGEF at the midzone and perhaps also activating it conformationally [48]. The ultimate output of this midzone-localized signaling network is an intense but narrow zone of cortical RhoA activity, and consequently, an equatorial cleavage furrow [18].

**Figure 6:**
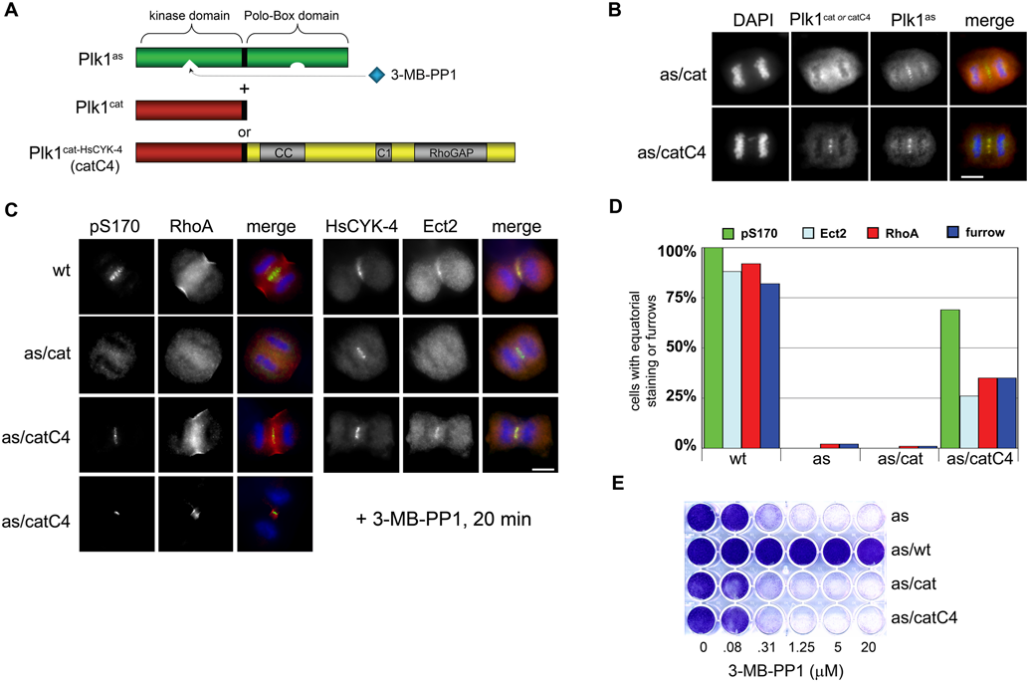
Recruiting Plk1 to the midzone via a PBD-independent mechanism reactivates the Ect2-RhoA network and induces equatorial cleavage furrows. (A) Plk1^as^ cells were transduced with retroviruses expressing either the catalytic domain of Plk1 alone (Plk1^cat^) or the same domain fused to HsCYK-4 (Plk1^catC4^) as FLAG-tagged proteins. (B) Plk1^catC4^ is recruited to the midzone during anaphase. Immunofluorescence microscopy was performed as in Figure 1B, except that anti-FLAG antibodies were used. Scale bar, 10 µm. (C) Plk1^catC4^ can activate the Ect2-RhoA network. Cells of each genotype were synchronized with monastrol, released, and treated with 3-MB-PP1 for 20 min. Phosphorylation of pS170, Ect2 recruitment, cortical RhoA recruitment, and incorporation of Citron kinase into the contractile ring were assessed by immunofluorescence microscopy. Scale bar, 10 µm. (D) The percentage of cells with equatorially localized proteins or ingressing furrows was quantified for each genotype. (E) Plk1^catC4^ confers cytokinesis-specific rescue of Polo kinase function. Cells were cultured in the presence of 3-MB-PP1 for 8 d and stained with crystal violet to assess growth.

This model not only explains why cytokinesis initiation is disrupted when Plk1's kinase activity is spatially dysregulated due to mutation of the PBD (Figure 1), but also predicts that one should be able to bypass this defect by targeting the kinase to the midzone and allowing it to phosphorylate HsCYK-4 via a PBD-independent mechanism. To test this prediction, we constructed retroviruses that express either the catalytic domain of Plk1 alone (Plk1^cat^) or the same domain fused to the N terminus of HsCYK-4 (an allele we term Plk1^catC4^) and introduced these constructs into Plk1^as^ cells (Figure 6A and Figure S1). During anaphase, Plk1^cat^ was found throughout the cell, while Plk1^catC4^ accumulated specifically at the midzone (Figure 5B). When Plk1^as/cat^ cells were challenged with 3-MB-PP1, S170 phosphorylation, Ect2 recruitment, equatorial RhoA activation, and furrow induction were all strongly inhibited (Figure 6C, second row, and Figure 6D). In contrast, a significant fraction of Plk1^as/catC4^ cells executed all three aspects of cytokinesis initiation and formed deep cleavage furrows (Figure 6C, bottom rows). This functional rescue was particularly striking given that the Plk1^catC4^ fusion was expressed at substantially lower levels than the untethered kinase domain (Figure S1). Importantly, the complementing activity of the Plk1^catC4^ allele was limited to cytokinesis onset, as other essential functions of Plk1 were not restored (Figure 6E).

**Figure 7:**
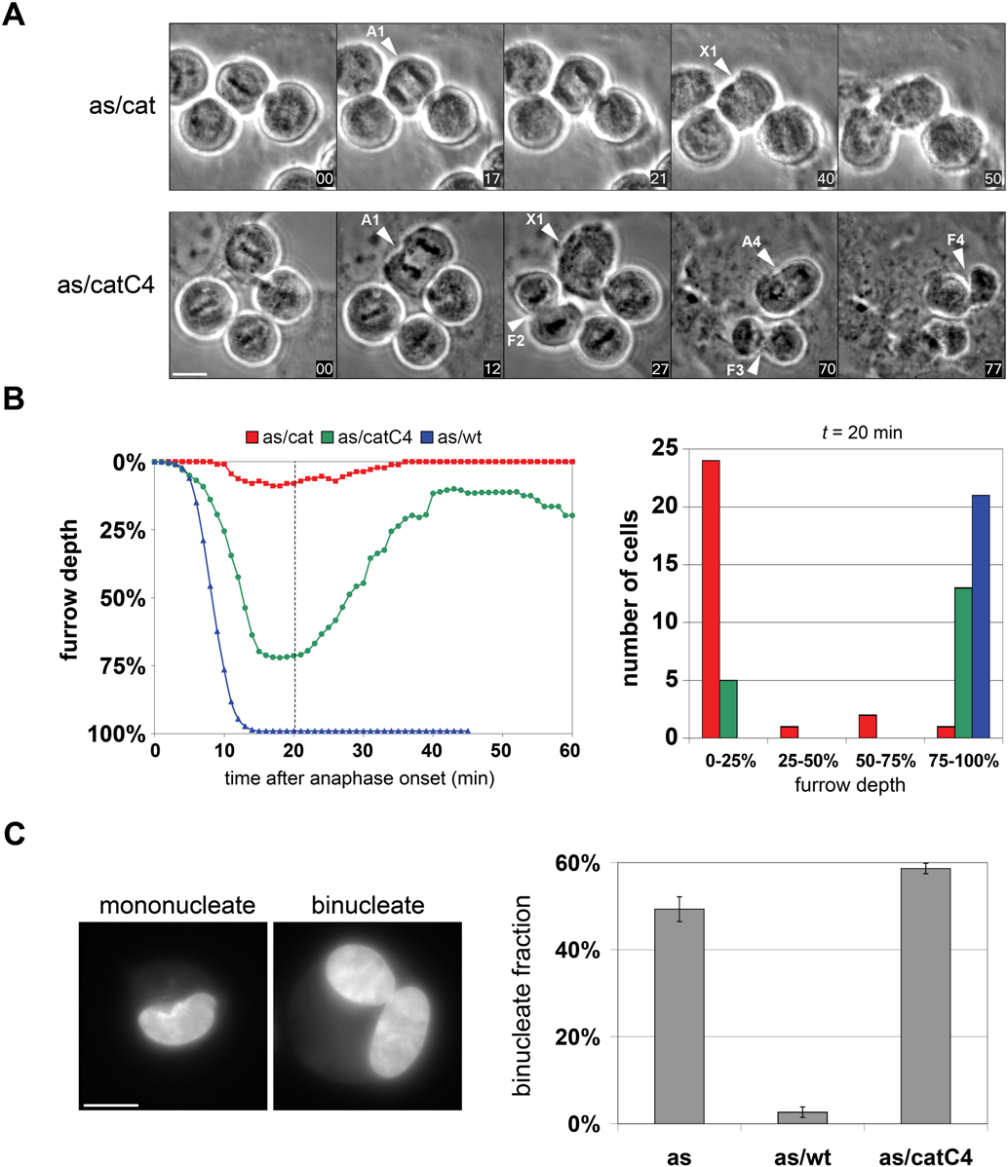
Tethering Plk1 to the centralspindlin complex rescues the initiation but not the completion of cytokinesis. (A) Monastrol-synchronized cells were released for 60 min into drug-free medium, followed by addition of 3-MB-PP1 and initiation of timelapse imaging (time 00). Examples of cells that traversed anaphase (A) and either formed equatorial furrows (F) or failed to do so (X) are indicated (see also Videos S1 and S2). Scale bar, 10 µm. (B) Biphasic furrow dynamics in Plk1^as/catC4^ cells. At least 18 cells of each genotype were imaged as above, and the average depth of the furrow relative to the initial equatorial diameter at anaphase onset was calculated. Furrow trajectories in individual cells are provided in Figure S6. (C) Cytokinesis ultimately fails in Plk1^as/catC4^ cells. Cells were synchronized in prometaphase by monastrol block and release as above. Thirty minutes after monastrol release, 3-MB-PP1 was added to 10 µM for an additional 90 min to inhibit Plk1^as^ activity during mitotic exit. Adherent cells were collected by trypsinization, fixed and stained with Hoechst 33352, and analyzed microscopically to determine the fraction of binucleated cells in each population (*n* = 3 determinations, 100 cells/sample; values are reported as means ± SEM). Scale bar, 10 µm.

To confirm and extend these results, we quantitatively analyzed cleavage furrow dynamics by time-lapse videomicroscopy (Figure 7 and Figure S6). Plk1^as/cat^ cells treated with 3-MB-PP1 either lacked furrows altogether (*n* = 21 cells; Figure 7A) or formed weak transient furrows that quickly collapsed (*n* = 7). In contrast, 66% of 3-MB-PP1-treated Plk1^as/catC4^ cells (*n* = 12 of 18 cells; Figure 7A) formed deep equatorial furrows, confirming rescue of cytokinesis onset. However, unlike wild-type furrows (Plk1^as/wt^ cells; *n* = 21, Figure 7B), these reconstituted furrows were unable to progress through abscission and ultimately regressed by the end of telophase, producing binucleate cells (Figure 7C). These results demonstrate chemical genetic separation of Plk1's roles during the early and late stages of cytokinesis and are consistent with recent data showing that Plk1 binds and phosphorylates other midzone- and midbody-specific proteins that are required to complete (but not initiate) cell division [35],[36],[44],[51],[52].

## Discussion

Cytokinesis must be temporally and spatially coordinated with chromosome segregation in order to avoid genome polyploidization, centrosome amplification, and chromosome breakage [4],[53]. In stem cell niches, the plane of division must also be correctly oriented relative to other asymmetrically distributed determinants of cell fate [54]. Conversely, defects in cytokinesis increase the likelihood of carcinogenesis [5], vividly highlighting the need to develop a detailed understanding of this process. While individual components of the cytokinetic apparatus are increasingly well annotated, our knowledge of how these components interact with one another at the systems level remains fragmentary [2]. In this study, we show that the self-regulated recruitment of Plk1 to the spindle midzone and its phosphorylation of the HsCYK-4 subunit of centralspindlin encode positive cues for cleavage furrow formation, thus providing new mechanistic insight into how mammalian cell division is regulated.

First, by manipulating Plk1 geometry in anaphase cells, we define a clear requirement for Plk1 activity at the midzone prior to recruitment of Ect2, cortical up-regulation of the RhoA GTPase, and contractile ring assembly. Although Plk1's PBD-dependent localization to the spindle midzone has long been appreciated [30],[34],[55], conducting a direct test of whether (and how) this spatial regulation contributes to the initial events in cell division has been virtually impossible, as cells expressing only PBD-deficient alleles of Plk1 are incapable of satisfying the spindle checkpoint and entering anaphase [33]. As an indirect alternative, RNAi-mediated depletion of individual Plk1 docking partners such as PRC1 [12],[36],[37] was used to block Plk1 enrichment at the midzone. Because such cells continue to form furrows, it was concluded that the midzone-associated pool of Plk1 plays no significant role upstream of the Ect2-RhoA network and contractile ring assembly [1],[37]–[39]. Based on the results obtained here, we suggest instead that such depletions did not abolish the full spectrum of PBD-dependent positive-feedback loops at the spindle midzone. In support of this view, Plk1 eventually accumulates on subcortical interzonal MTs in PRC1-depleted cells [36], presumably via redundant interactions with other MT-binding proteins such as MKLP2 [56] and HsCYK-4 (Figure 2). It is also possible that the lack of a bona fide spindle midzone in PRC1-depleted cells actually enhances furrowing by increasing the number of interzonal MTs that contact the equatorial cortex [57].

In contrast, by using chemical genetics to inactivate or bypass all PBD-dependent positive-feedback loops in anaphase cells, we are now able to show that Plk1 self-organization at the midzone is essential for cytokinesis onset in human cells. This regulatory mechanism is conceptually similar to other positive-feedback loops that govern Cdk1 activation at the G1/S and G2/M transitions [58],[59] and cyclin B and securin proteolysis at anaphase onset [60],[61]. However, it is unique in that it works by concentrating Plk1 near its midzone-specific substrates, rather than by up-regulating Plk1's activity throughout the cytoplasm. Nevertheless, like other positive-feedback loops, Plk1 self-organization makes cytokinesis more switchlike—that is, more rapid and synchronous relative to other late mitotic events—than would otherwise be the case. Indeed, in cells expressing the active but unanchored kinase fragment (Plk1^cat^), furrows were not only rarer but also shallower and more temporally heterogeneous than those induced by midzone-localized versions of Plk1 (Figure 7 and Figure S6). We suggest that Plk1-mediated cytokinesis synchrony could play an important role in ensuring that cells initiate and complete division before their license to do so expires, typically around 30 to 60 min after anaphase onset [62]–[64].

Like Plk1, Aurora B also relocalizes onto the spindle midzone at anaphase onset, resulting in an axial phosphorylation gradient that can be detected using a fluorescence resonant energy transfer (FRET)-based biosensor [65]. Although a Plk1-generated gradient has yet to be detected by this method, our results show that Plk1's association with the midzone predicts whether or not an equatorial cleavage furrow will form (Figures 6 and 7). If an anaphase Plk1 gradient does exist, its detection may require the development of newer biosensors that can engage in PBD-mediated positive feedback and thus mimic the behavior of Plk1's anaphase-specific substrates.

**Figure 8:**
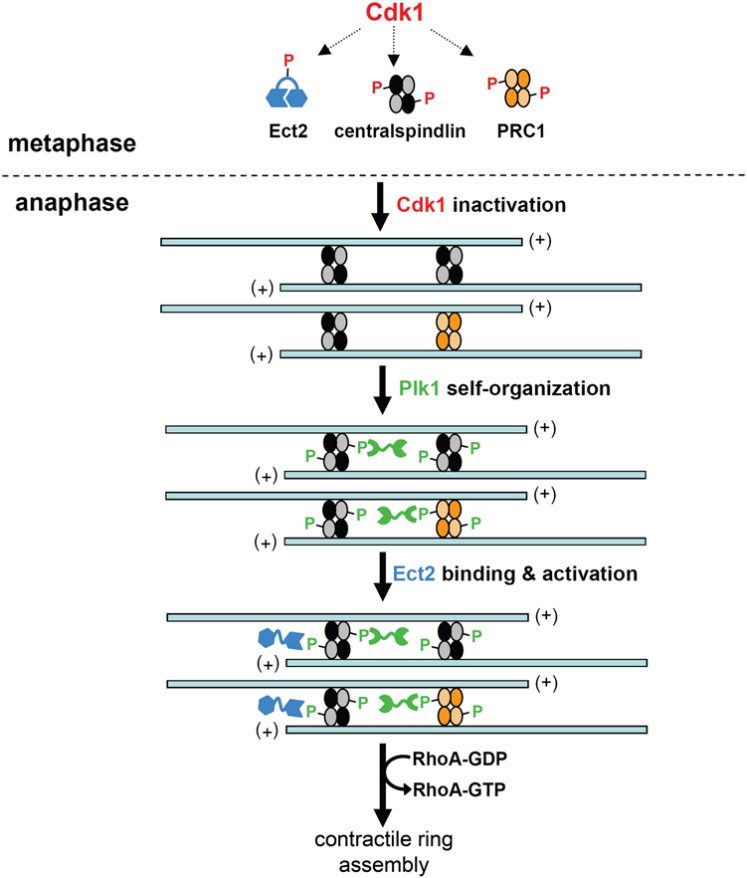
A model for Plk1-mediated generation of the midzone-derived signal for cell division. In prometaphase, high Cdk1 levels maintain Ect2, centralspindlin, and PRC1 in a functionally inactive state. Upon Cdk1 inactivation at anaphase onset, the MKLP1 subunit of centralspindlin and PRC1 are dephosphorylated and thus able to bind and bundle antiparallel MT arrays to form the spindle midzone [12],[13]. At the same time, Plk1 is released from its early mitotic substrates by dephosphorylation of Cdk1-primed PBD docking sites, and inhibitory phosphorylation of Ect2 at Cdk1 sites is also reversed [14]. Plk1 then rapidly self-organizes onto the midzone via PBD- and activity-dependent positive-feedback loops involving multiple substrates, including PRC1 [36], MKLP2 [56], and HsCYK-4 (this study). Furthermore, in the case of HsCYK-4, Plk1 phosphorylation at S157 also creates a docking site for the tandem BRCT repeats of Ect2, thereby targeting the RhoGEF to the midzone and perhaps stimulating its activity [48]. The resulting increase in RhoA-GTP at the adjacent equatorial cortex promotes focal recruitment and activation of RhoA effectors needed for actin polymerization and myosin II motor activity within the contractile ring [2],[3].

We have also demonstrated that HsCYK-4 is an in vivo substrate of Plk1 at the spindle midzone, and that this phosphorylation allows HsCYK-4 to be recognized by the tandem BRCT repeats at the N terminus of Ect2. These results explain why Ect2 becomes mislocalized in cells treated with Plk1 inhibitors [24],[27]–[29], and together with the contractile ring assembly defect in cells expressing non-phosphorylatable HsCYK-4 (Figure 4), they suggest that HsCYK-4 is Plk1's major rate-limiting substrate upstream of RhoA. Curiously, this pronounced phenotype contrasts with the behavior of cells expressing dominant-negative Ect2 fragments, as the latter activate RhoA and ingress their furrows normally [16]. By their very nature, dominant-negative experiments are difficult to judge for penetrance and specificity, as it can always be argued that the endogenous protein's function has been inadequately antagonized, or that another factor (in this case, a hypothetical negative regulator of furrowing) was also titrated. However, a more interesting notion is that HsCYK-4 mutations prevent not only Ect2 recruitment to the midzone but also relief of its auto-inhibition (Figure 8) [48], whereas dominant-negative Ect2 fragments allow the RhoGEF to cycle on and off HsCYK-4 in an activated state. Further experiments will be required to test this idea and clarify whether additional modes of Ect2 regulation operate in parallel with the mechanisms described here.

In summary, our findings indicate that Plk1 generates the midzone-encoded stimulus for cell division by priming two structurally distinct phosphopeptide-binding modules within Ect2 and Plk1 itself, thereby ensuring the robustness and fidelity of this process (Figure 8). Curiously, although the proximal and distal elements of this cytokinesis network (Plk1 and RhoA) are universally conserved [66],[67], the intermediate components that couple this network to MTs (centralspindlin and Ect2) are either missing or lack cognate phosphopeptide-binding domains in unicellular organisms such as budding and fission yeast, which establish their division sites without reference to spindle geometry [68]. It is tempting to speculate that the MT dependence of cytokinesis in metazoans is specifically related to the evolutionary history of these regulators of cell division.

Finally, we note that although genetic and pharmacologic probes are increasingly available for many cell cycle regulators, including Plk1, these tools typically involve substantial tradeoffs between temporal and spatial resolution. For instance, RNAi-based knockdown/addback methods allow researchers to deplete or relocalize a given enzyme, but require days to take effect. Conversely, pharmacologic agents have much faster kinetics of onset, but their high diffusibility limits their usefulness in selectively activating or inhibiting an enzyme at a defined subcellular location. Furthermore, distinguishing between the on-target and off-target effects of such compounds can be quite difficult. As illustrated here, chemical genetics can overcome these limitations and provide novel insights that are inaccessible via other routes. Given existing methods for gene replacement, similar systems can now be envisioned for most or all of the 600 kinases in the mouse and human genomes, providing powerful tools for both fundamental physiologic studies and preclinical evaluation of these enzymes as therapeutic targets.

## Materials and Methods

### Cell Culture Procedures

All lines were propagated in the following media supplemented with 10% fetal bovine serum and 100 units/ml penicillin-streptomycin: HeLa and Phoenix retroviral packaging lines, Dulbecco's modified Eagle's medium (DMEM); HCT116 colorectal carcinoma cells, McCoy's 5A medium; and hTERT-RPE1 retinal pigment epithelial cells, 1∶1 mixture of DMEM and Ham's F-12 medium supplemented with 2.5 mM L-glutamine. Plasmid transfections were performed on cells at 50–70% confluence using Fugene 6 (Roche) and 8 µg total DNA per 10^7^ cells. For growth assays, cells were split 1∶40 into 12-well plates, and after 24 h, the indicated concentration of 3-MB-PP1 was added to the medium. When untreated control wells reached confluence (typically day 8), the medium was aspirated, and adherent cells visualized by fixation and staining with crystal violet in buffered formalin. The generation of Plk1^as^ cells was previously described [24]. To enable G418 selection, the *FRT-neo^R^-FRT* cassette used to disrupt the endogenous *PLK1* locus in these cells was excised via transient transfection with a FLP recombinase plasmid. G418-sensitive clones obtained in this manner were in all other respects indistinguishable from the original Plk1^as^ cells.

### Molecular Biology and Retroviral Transgenesis

Plasmid mutagenesis was performed using the QuikChange XL II kit (Stratagene), and all inserts were fully sequenced to verify their integrity. For stable retroviral transduction, inserts were cloned into pQCXIN or pQCXIX (Clontech), and the resulting constructs cotransfected with a VSV-G envelope plasmid into Phoenix cells. Fresh medium was applied 24 h post-transfection and harvested 24 h later, clarified by centrifugation and filtration through a 0.4 µm membrane to remove cell debris, and diluted 1∶1 with complete medium containing 20 µg/ml polybrene. Target cells were infected at 40–60% confluence for 24–48 h, then selected with 0.4 mg/ml G418 for 7–10 d, or alternatively expanded for FACS purification based on GFP or mCherry positivity. In some cases, these polyclonal transductants were further purified by limiting dilution to obtain individual clones.

### Protein Expression and Purification

Human Plk1 was expressed as a hexahistidine-tagged polypeptide in Sf9 cells using the FastBAC system (Invitrogen), purified by nickel affinity chromatography, and dialyzed into storage buffer containing 50 mM Tris, pH 8.0, 150 mM NaCl, and 10% glycerol. Recombinant HsCYK-4N fragments were expressed as chitin-binding domain (CBD) and hexahistidine fusions in the Rosetta (DE3) *Escherichia coli* strain (Novagen) and purified by nickel affinity chromatography.

### Kinase Reactions and Chemicals

Kinase reactions were performed as previously described [24]. For radiolabeling experiments, [γ-^32^P]ATP was diluted with 100 µM cold ATP and incubated for 30 min at 30°C. For nonradioactive reactions, 1 mM cold ATP was used. Chemicals used in this study include 3-MB-PP1 (10 µM or as indicated), BI 2536 (200 nM), BTO-1 (Sigma, 50 µM), dithiobis[succinimidylpropionate] (DSP, Pierce, 1 mg/ml), monastrol (Calbiochem, 100 µM), nocodazole (Sigma, 0.2 mg/ml), RO-3306 (Calbiochem, 10 µM), S-trityl-L-cysteine (Acros Organics, 5 µM), and thymidine (Calbiochem, 2.5 mM).

### Mass Spectrometry

The in vitro kinase reaction of Plk1 and HsCYK-4N was boiled in reducing sample buffer containing β-mercaptoethanol, separated by SDS-PAGE, and visualized by SYPRO Ruby staining (Bio-Rad). The HsCYK-4 band was excised from the gel and cut in half vertically. One-half was digested overnight at 37°C with an excess of sequencing grade trypsin, the other with Glu-C, in order to generate peptides with maximal coverage. Peptides were extracted from the gel slices with 50% acetonitrile/0.1% formic acid and concentrated in a Speed-Vac. The quenched tryptic and Glu-C digests were mixed together and analyzed with an automated nano LC/MS/MS system, using a 1200 series autosampler and nano pump (Agilent Technologies) coupled to an LTQ XL ion trap mass spectrometer (Thermo Electron). Peptides were eluted from a 75 mm × 10 cm PicoFrit (New Objective) column packed with 5 µm Magic C-18AQ reversed phase beads (Michrom Bioresources) using a 70-min acetonitrile/0.1% formic acid gradient at a flow rate of 250 nl/min to yield ∼25-s peak widths. Data-dependent LC/MS/MS spectra were acquired in 3-s cycles; each cycle was of the following form: one full MS scan followed by eight MS/MS scans in the ion trap on the most abundant precursor ions subject to dynamic exclusion. Sites of phosphorylation were established by searching the MS/MS spectra with the Spectrum Mill software package (Agilent Technologies) using a database containing the HsCYK-4 sequence and allowing variable modification of Ser and Thr by phosphorylation. Labeled MS/MS spectra that demonstrate phosphorylation at Ser157, Ser170, Ser214, and Thr 260 are provided in Figure S2.

### RNA Interference

For RNAi experiments, six silent mutations were introduced into the target site (nt 1,294–1,312) of a validated HsCYK-4 siRNA duplex [14]. This siRNA-resistant open reading frame was then cloned downstream of EGFP in pQCXIN and used to stably transduce hTERT-RPE1 cells. To deplete endogenous HsCYK-4, cells were plated into 6-well dishes and transfected with 150 pmol siRNA and 5 µl Lipofectamine 2000 (Invitrogen). Twenty-four hours later, cells were trypsinized, resuspended in 5 ml antibiotic-free medium, and replated as follows: (i) one–twenty-fifth was plated into 4-well chamber slides (Labtek), synchronized with monastrol from 40–48 h post-transfection, released into monastrol-free medium for 50 min, and then fixed and processed for immunofluorescence microscopy; (ii) one-tenth was replated into a new 6-well dish, then collected 24 h later for determination of the fraction of mononucleated versus binucleated cells by Hoechst staining and microscopy; (iii) the remainder was passaged to a T-25 flask and collected 24 h later for SDS-PAGE and immunoblotting.

### Immunofluorescence and Live-Cell Microscopy

Cells were plated in 4-well chamber slides and grown to ∼70% density, synchronized in prometaphase with an 8-h treatment with monastrol, then washed and released into monastrol-free medium. Using time-lapse videomicroscopy, we determined that the initial wave of anaphase cells can be detected approximately 50 min after monastrol washout. Thus, where indicated 3-MB-PP1 was added 30 min after washout and maintained for a 20-min interval prior to fixation with either 10% ice-cold trichloroacetic acid for 10 min (to detect activated RhoA), 4% paraformaldehyde for 15 min (Citron-kinase), or 100% ice-cold methanol for 15 min to overnight (all other antigens). Fixed cells were washed once in phosphate-buffered saline (PBS) and blocked for 30 min in 3% bovine serum albumin (BSA) and 0.1% Triton X-100 in PBS (PBSTx+BSA). Primary antibodies were applied in the same buffer for 2 h at room temperature, followed by three washes in PBSTx. Alexa 488-, 594-, or 647-conjugated goat secondary antibodies were then applied in a similar manner, followed by DAPI counterstaining and mounting in Prolong Plus antifade medium (Invitrogen). To detect the goat anti-HsCYK-4 antibody, an Alexa-coupled donkey anti-goat antibody was used as the secondary antibody, prior to incubation with goat-derived secondary antibodies. Antibody dropout experiments were included to verify specificity of staining and lack of crosstalk. Equatorial enrichment of antigens was ascertained by qualitative classification of cells in a blinded manner. Image acquisition was performed on a Nikon TE2000 inverted microscope equipped with 10× and 40× long working distance and 100× oil objectives, single-bandpass excitation and emission filters, Hamamatsu ORCA ER camera, a temperature-controlled stage enclosure with CO_2_ support (Solent Scientific), and MetaMorph software (Molecular Devices). Panels were cropped and assembled into figures using Photoshop CS2 (Adobe).

### Immunoprecipitation and Blotting

For coprecipitation of endogenous HsCYK-4-associated proteins (Figure 2C), HeLa cells were split into T-75 flasks at 20% density in the presence of thymidine. After 24 h, cells were washed twice with Hank's balanced salt solution (HBSS) and released into fresh medium. After an additional 12 h, thymidine was added. Fifteen hours later, cells were washed twice with HBSS and released into fresh medium containing nocodazole. After 12 h, cells were washed twice and transferred into nocodazole-free medium. At each timepoint, DSP was added for 10 min to stabilize HsCYK-4 associated proteins, followed by quenching in 0.1 M Tris pH 8.0 for 10 min. Cells were collected and lysed in buffer (50 mM HEPES pH 7.5, 100 mM NaCl, 0.5% NP-40, 10% glycerol) containing phosphatase inhibitors (10 mM sodium pyrophosphate, 5 mM β-glycerolphosphate, 50 mM NaF, 0.3 mM Na_3_VO_4_), protease inhibitors (1mM PMSF, 1× protease inhibitor cocktail (Sigma)), and 1 mM dithiothreitol. Two micrograms anti-HsCYK-4 antibody or control goat IgG was added to 1 mg extract on ice, followed by retrieval of immune complexes with a 1∶1 mixture of protein A- and protein G-coupled Sepharose beads. After extensive washes, bead-bound proteins were boiled in 1× Laemmli buffer containing β-mercaptoethanol to reverse DSP-generated crosslinks prior to SDS-PAGE and immunoblotting. A similar protocol was used for all other immunoprecipitation experiments, except that DSP treatment was omitted. To specifically inhibit the 9E10 anti-myc antibody, a 100-fold excess of competitor peptide (EQKLISEEDL; Anaspec) was added on ice for 1 h before immunoprecipitation. Far-Western blotting with the GST-PBD fusion protein was performed as described [44].

### Antibodies

Phosphoepitope-specific antibodies to HsCYK-4 were generated in rabbits through immunization of pS157- or p170-specific phosphopeptides (Active Motif). Sera were screened for phosphoselectivity by peptide dot-blotting and immunofluorescence microscopy. For affinity purification, phosphopeptides were coupled to a solid-phase support via maleimide chemistry. Anti-pS170 (rabbit 8044) was used for immunofluorescence (1∶500) and Western blotting (1∶1000). Anti-pS157 (rabbit 8041) was used for immunofluorescence (1∶100). Other antibodies used in this study: Chitin-binding domain (CBD; New England Biolabs) 1∶1000 WB; Citron kinase (Becton Dickinson) 1∶200 IF; Ect2 [14] 1∶350 IF; Ect2 sc-1005 (Santa Cruz) 1∶1,000 WB; FLAG M2 (Sigma) 1∶500 IF, 1∶2,000 WB; GST-HRP sc-459 (Santa Cruz), 1∶5,000; RACGAP1 (Abnova) 1∶1,000 WB; RACGAP1 (GeneTex) 1∶500 IF, IP; GFP clone 3E6 (Invitrogen) 1∶1,000 IF; dsRed (detects mCherry; Clontech) 1∶1,000 WB, 1∶1,000 IF; Myc 9E10 (MSKCC Hybridoma Core Facility) IP; myc 9E10-HRP conjugate (Roche) 1∶5,000 WB; RhoA sc-418 (Santa Cruz) 1∶200 IF; Pds1 DCS-280 (Neomarkers) 1∶500 WB; Plk1 N-terminal 06-813 (Millipore) 1∶500 WB; Plk1 F-8 (Santa Cruz) 1∶1,000 WB; α-tubulin sc-5286 (Santa Cruz), 1∶2,000 WB.

## Supporting Information

Figure S1 **Expression of Plk1 alleles.** Whole-cell extracts from cells of the indicated genotypes were resolved by SDS-PAGE and immunoblotted with antibodies specific for Plk1 (top panel) or mCherry (bottom panel). Note that mCherry- and EGFP-fused Plk1 proteins differ by only four amino acids in length and thus comigrate. Asterisk indicates a minor breakdown product of mCherry-Plk1 fusion proteins. A nonspecific band used as a loading control is also indicated.
(0.19 MB PDF)

Figure S2 **Identification of HsCYK-4 phosphorylation sites by mass spectrometry.** A recombinant HsCYK-4N fragment was phosphorylated with purified Plk1 and then processed for tandem mass spectrometry as detailed in the Methods. MS/MS spectra of peptides containing the indicated sites are shown with b-ions in blue and y-ions in red. Ions indicating neutral loss of phosphate from the precursor are shown in green.
(0.97 MB PDF)

Figure S3 **Analysis of pS170 in synchronized HeLa cells.** HeLa cells were synchronized by sequential thymidine and nocodazole blocks and released into drug-free medium at time 0. After sampling at various timepoints, whole-cell extracts were prepared and immunoprecipitated with anti-HsCYK-4 antibodies, then immunoblotted to detect either S170 phosphorylation or total HsCYK-4.
(0.14 MB PDF)

Figure S4 **Localization of GFP-HsCYK-4 fusions and validation of the pS170 antibody.** (A) RPE cells stably expressing siRNA-resistant GFP-HsCYK-4^wt^ or GFP-HsCYK-4^5A^ were stained with a GFP-specific monoclonal antibody and analyzed by fluorescence microscopy. Note comparable incorporation of both GFP-tagged HsCYK-4 alleles into the spindle midzone. Scale bar, 10 µm. (B) HeLa cells were transiently transfected with constructs expressing wild-type, S157A, or S170A versions of FLAG-HsCYK-4 and arrested in mitosis with nocodazole. Extracts were immunoprecipitated with anti-FLAG antibody-Sepharose beads, resolved by SDS-PAGE, and immunoblotted with anti-pS170 antibody (top) or anti-FLAG antibody (bottom). Asterisk denotes crossreaction with immunoglobulin heavy chain. (C) RPE cells were transfected with HsCYK-4 or mock siRNAs, either before (top two rows) or after (bottom two rows) stable transduction with retroviruses expressing siRNA-resistant GFP-HsCYK-4 or GFP-HsCYK-4^S170A^. Cells were stained with goat antibodies to HsCYK-4 (green) and rabbit anti-pS170 antibodies (red). Note that the S170A transgene restores HsCYK-4 but not pS170. Scale bar, 10 µm.
(0.79 MB PDF)

Figure S5 **Validation of the pS157 antibody.** (A) Decreasing amounts of phosphorylated (pS157) or unphosphorylated (S157) peptides were spotted on PVDF membranes and probed with anti-pS157 antibodies. (B) HeLa cells were transiently transfected with plasmids expressing wild-type or S157A versions of FLAG-HsCYK-4 (or mock-transfected as a negative control) and arrested in mitosis with nocodazole. Extracts were immunoprecipitated with anti-FLAG antibody-Sepharose beads, resolved by SDS-PAGE, and immunoblotted with anti-pS157 antibody (top) or anti-FLAG antibody (bottom). Asterisk denotes crossreaction with immunoglobulin heavy chain. (C) RPE cells were transfected with HsCYK-4 or mock siRNAs, either before (top two rows) or after (bottom two rows) stable transduction with retroviruses expressing siRNA-resistant GFP-HsCYK-4 or GFP-HsCYK-4^S157A^. Cells were stained with goat antibodies to HsCYK-4 (green) and rabbit anti-pS157 antibodies (red). Note that the S157A transgene restores HsCYK-4 but not pS157. Scale bar, 10 µm.
(0.78 MB PDF)

Figure S6 **Phosphorylation of serine 157 by Plk1 promotes assembly of the Ect2/HsCYK-4 complex.** HeLa cells were transiently transfected with plasmids expressing FLAG epitope-tagged HsCYK-4 (wild-type, S157A, and S170A) and myc epitope-tagged Ect2 (myc-Ect2). Twenty-four hours after transfection, cells were arrested in mitosis for 15 hours with 5 µM S-trityl-L-cysteine [70] (+ 200 nM BI 2536 where indicated), then treated with the Cdk1-specific inhibitor RO-3306 [71] for 20 min to induce mitotic exit. Whole-cell extracts were immunoprecipitated with anti-myc antibodies, resolved by SDS-PAGE, and immunoblotted with anti-FLAG and anti-myc antibodies.
(0.34 MB PDF)

Figure S7 **Individual furrow trajectories in Plk1^as/wt^, Plk1^as/cat^, and Plk1^as/catC4^ cells.** Cells of each genotype were released from a monastrol block and imaged by phase-contrast videomicroscopy in the presence of 10 µM 3-MB-PP1. The time at which each cell entered anaphase was noted and set as time 0, and the depth of the cleavage furrow was measured at each subsequent frame (1/min) until the cell exited mitosis and adopted a flat morphology. Furrow trajectories are displayed as heat maps to facilitate visual inspection of the dataset.
(0.89 MB PDF)

Video S1 **Furrow formation is defective in Plk1^as/cat^ cells.** This movie corresponds to the upper panel in Figure 7A.
(3.26 MB MOV)

Video S2 **Furrow formation is rescued in Plk1^as/catC4^ cells.** This movie corresponds to the lower panel in Figure 7A.
(7.11 MB MOV)

## Abbreviations

MT: microtubules
PBD: Polo-box domain
RPE: retinal pigment epithelium

## References

[1] Barr FA, Gruneberg U (2007). Cytokinesis: placing and making the final cut.. Cell.

[2] Eggert US, Mitchison TJ, Field CM (2006). Animal cytokinesis: from parts list to mechanisms.. Annu Rev Biochem.

[3] Glotzer M (2005). The molecular requirements for cytokinesis.. Science.

[4] Ganem NJ, Storchova Z, Pellman D (2007). Tetraploidy, aneuploidy and cancer.. Curr Opin Genet Dev.

[5] Fujiwara T, Bandi M, Nitta M, Ivanova EV, Bronson RT (2005). Cytokinesis failure generating tetraploids promotes tumorigenesis in p53-null cells.. Nature.

[6] Rappaport R (1996). Cytokinesis in Animal Cells.

[7] Alsop GB, Zhang D (2003). Microtubules are the only structural constituent of the spindle apparatus required for induction of cell cleavage.. J Cell Biol.

[8] Rappaport R (1961). Experiments concerning the cleavage stimulus in sand dollar eggs.. J Exp Zool.

[9] Bringmann H, Hyman AA (2005). A cytokinesis furrow is positioned by two consecutive signals.. Nature.

[10] Murthy K, Wadsworth P (2008). Dual role for microtubules in regulating cortical contractility during cytokinesis.. J Cell Sci.

[11] Chen W, Foss M, Tseng KF, Zhang D (2008). Redundant mechanisms recruit actin into the contractile ring in silkworm spermatocytes.. PLoS Biol.

[12] Mollinari C, Kleman JP, Jiang W, Schoehn G, Hunter T (2002). PRC1 is a microtubule binding and bundling protein essential to maintain the mitotic spindle midzone.. J Cell Biol.

[13] Mishima M, Pavicic V, Gruneberg U, Nigg EA, Glotzer M (2004). Cell cycle regulation of central spindle assembly.. Nature.

[14] Yuce O, Piekny A, Glotzer M (2005). An ECT2-centralspindlin complex regulates the localization and function of RhoA.. J Cell Biol.

[15] Zhao WM, Fang G (2005). MgcRacGAP controls the assembly of the contractile ring and the initiation of cytokinesis.. Proc Natl Acad Sci U S A.

[16] Chalamalasetty RB, Hummer S, Nigg EA, Sillje HH (2006). Influence of human Ect2 depletion and overexpression on cleavage furrow formation and abscission.. J Cell Sci.

[17] Kamijo K, Ohara N, Abe M, Uchimura T, Hosoya H (2006). Dissecting the role of Rho-mediated signaling in contractile ring formation.. Mol Biol Cell.

[18] Bement WM, Benink HA, von Dassow G (2005). A microtubule-dependent zone of active RhoA during cleavage plane specification.. J Cell Biol.

[19] Somers WG, Saint R (2003). A RhoGEF and Rho family GTPase-activating protein complex links the contractile ring to cortical microtubules at the onset of cytokinesis.. Dev Cell.

[20] Vagnarelli P, Earnshaw WC (2004). Chromosomal passengers: the four-dimensional regulation of mitotic events.. Chromosoma.

[21] Petronczki M, Lenart P, Peters JM (2008). Polo on the Rise-from Mitotic Entry to Cytokinesis with Plk1.. Dev Cell.

[22] Randall CL, Burkard ME, Jallepalli PV (2007). Polo kinase and cytokinesis initiation in Mammalian cells: harnessing the awesome power of chemical genetics.. Cell Cycle.

[23] Lenart P, Petronczki M, Steegmaier M, Di Fiore B, Lipp JJ (2007). The small-molecule inhibitor BI 2536 reveals novel insights into mitotic roles of polo-like kinase 1.. Curr Biol.

[24] Burkard ME, Randall CL, Larochelle S, Zhang C, Shokat KM (2007). Chemical genetics reveals the requirement for Polo-like kinase 1 activity in positioning RhoA and triggering cytokinesis in human cells.. Proc Natl Acad Sci U S A.

[25] McInnes C, Mazumdar A, Mezna M, Meades C, Midgley C (2006). Inhibitors of Polo-like kinase reveal roles in spindle-pole maintenance.. Nat Chem Biol.

[26] Peters U, Cherian J, Kim JH, Kwok BH, Kapoor TM (2006). Probing cell-division phenotype space and Polo-like kinase function using small molecules.. Nat Chem Biol.

[27] Santamaria A, Neef R, Eberspacher U, Eis K, Husemann M (2007). Use of the novel Plk1 inhibitor ZK-thiazolidinone to elucidate functions of Plk1 in early and late stages of mitosis.. Mol Biol Cell.

[28] Brennan IM, Peters U, Kapoor TM, Straight AF (2007). Polo-like kinase controls vertebrate spindle elongation and cytokinesis.. PLoS ONE.

[29] Petronczki M, Glotzer M, Kraut N, Peters JM (2007). Polo-like kinase 1 triggers the initiation of cytokinesis in human cells by promoting recruitment of the RhoGEF Ect2 to the central spindle.. Dev Cell.

[30] Golsteyn RM, Mundt KE, Fry AM, Nigg EA (1995). Cell cycle regulation of the activity and subcellular localization of Plk1, a human protein kinase implicated in mitotic spindle function.. J Cell Biol.

[31] Elia AE, Cantley LC, Yaffe MB (2003). Proteomic screen finds pSer/pThr-binding domain localizing Plk1 to mitotic substrates.. Science.

[32] Elia AE, Rellos P, Haire LF, Chao JW, Ivins FJ (2003). The molecular basis for phosphodependent substrate targeting and regulation of Plks by the Polo-box domain.. Cell.

[33] Hanisch A, Wehner A, Nigg EA, Sillje HH (2006). Different Plk1 functions show distinct dependencies on Polo-Box domain-mediated targeting.. Mol Biol Cell.

[34] Seong YS, Kamijo K, Lee JS, Fernandez E, Kuriyama R (2002). A spindle checkpoint arrest and a cytokinesis failure by the dominant-negative polo-box domain of Plk1 in U-2 OS cells.. J Biol Chem.

[35] Mollinari C, Kleman JP, Saoudi Y, Jablonski SA, Perard J (2005). Ablation of PRC1 by small interfering RNA demonstrates that cytokinetic abscission requires a central spindle bundle in mammalian cells, whereas completion of furrowing does not.. Mol Biol Cell.

[36] Neef R, Gruneberg U, Kopajtich R, Li X, Nigg EA (2007). Choice of Plk1 docking partners during mitosis and cytokinesis is controlled by the activation state of Cdk1.. Nat Cell Biol.

[37] D'Avino PP, Archambault V, Przewloka MR, Zhang W, Lilley KS (2007). Recruitment of Polo kinase to the spindle midzone during cytokinesis requires the Feo/Klp3A complex.. PLoS ONE.

[38] van Vugt MA, Medema RH (2005). Getting in and out of mitosis with Polo-like kinase-1.. Oncogene.

[39] van de Weerdt BC, Medema RH (2006). Polo-like kinases: a team in control of the division.. Cell Cycle.

[40] Ferrell JE (2002). Self-perpetuating states in signal transduction: positive feedback, double-negative feedback and bistability.. Curr Opin Cell Biol.

[41] Ubersax JA, Ferrell JE (2007). Mechanisms of specificity in protein phosphorylation.. Nat Rev Mol Cell Biol.

[42] van Vugt MA, van de Weerdt BC, Vader G, Janssen H, Calafat J (2004). Polo-like kinase-1 is required for bipolar spindle formation but is dispensable for anaphase promoting complex/Cdc20 activation and initiation of cytokinesis.. J Biol Chem.

[43] Steegmaier M, Hoffmann M, Baum A, Lenart P, Petronczki M (2007). BI 2536, a potent and selective inhibitor of polo-like kinase 1, inhibits tumor growth in vivo.. Curr Biol.

[44] Lowery DM, Clauser KR, Hjerrild M, Lim D, Alexander J (2007). Proteomic screen defines the Polo-box domain interactome and identifies Rock2 as a Plk1 substrate.. Embo J.

[45] Nousiainen M, Sillje HH, Sauer G, Nigg EA, Korner R (2006). Phosphoproteome analysis of the human mitotic spindle.. Proc Natl Acad Sci U S A.

[46] Olsen JV, Blagoev B, Gnad F, Macek B, Kumar C (2006). Global, in vivo, and site-specific phosphorylation dynamics in signaling networks.. Cell.

[47] Eda M, Yonemura S, Kato T, Watanabe N, Ishizaki T (2001). Rho-dependent transfer of Citron-kinase to the cleavage furrow of dividing cells.. J Cell Sci.

[48] Kim JE, Billadeau DD, Chen J (2005). The tandem BRCT domains of Ect2 are required for both negative and positive regulation of Ect2 in cytokinesis.. J Biol Chem.

[49] Manke IA, Lowery DM, Nguyen A, Yaffe MB (2003). BRCT repeats as phosphopeptide-binding modules involved in protein targeting.. Science.

[50] Yu X, Chini CC, He M, Mer G, Chen J (2003). The BRCT domain is a phospho-protein binding domain.. Science.

[51] Fabbro M, Zhou BB, Takahashi M, Sarcevic B, Lal P (2005). Cdk1/Erk2- and Plk1-dependent phosphorylation of a centrosome protein, Cep55, is required for its recruitment to midbody and cytokinesis.. Dev Cell.

[52] Litvak V, Argov R, Dahan N, Ramachandran S, Amarilio R (2004). Mitotic phosphorylation of the peripheral Golgi protein Nir2 by Cdk1 provides a docking mechanism for Plk1 and affects cytokinesis completion.. Mol Cell.

[53] Norden C, Mendoza M, Dobbelaere J, Kotwaliwale CV, Biggins S (2006). The NoCut pathway links completion of cytokinesis to spindle midzone function to prevent chromosome breakage.. Cell.

[54] Lin H (2008). Cell biology of stem cells: an enigma of asymmetry and self-renewal.. J Cell Biol.

[55] Arnaud L, Pines J, Nigg EA (1998). GFP tagging reveals human Polo-like kinase 1 at the kinetochore/centromere region of mitotic chromosomes.. Chromosoma.

[56] Neef R, Preisinger C, Sutcliffe J, Kopajtich R, Nigg EA (2003). Phosphorylation of mitotic kinesin-like protein 2 by polo-like kinase 1 is required for cytokinesis.. J Cell Biol.

[57] Kurasawa Y, Earnshaw WC, Mochizuki Y, Dohmae N, Todokoro K (2004). Essential roles of KIF4 and its binding partner PRC1 in organized central spindle midzone formation.. Embo J.

[58] Pomerening JR, Kim SY, Ferrell JE (2005). Systems-level dissection of the cell-cycle oscillator: bypassing positive feedback produces damped oscillations.. Cell.

[59] Skotheim JM, Di Talia S, Siggia ED, Cross FR (2008). Positive feedback of G1 cyclins ensures coherent cell cycle entry.. Nature.

[60] Holt LJ, Krutchinsky AN, Morgan DO (2008). Positive feedback sharpens the anaphase switch.. Nature.

[61] Visintin R, Craig K, Hwang ES, Prinz S, Tyers M (1998). The phosphatase Cdc14 triggers mitotic exit by reversal of Cdk-dependent phosphorylation.. Mol Cell.

[62] Canman JC, Hoffman DB, Salmon ED (2000). The role of pre- and post-anaphase microtubules in the cytokinesis phase of the cell cycle.. Curr Biol.

[63] Martineau SN, Andreassen PR, Margolis RL (1995). Delay of HeLa cell cleavage into interphase using dihydrocytochalasin B: retention of a postmitotic spindle and telophase disc correlates with synchronous cleavage recovery.. J Cell Biol.

[64] Straight AF, Cheung A, Limouze J, Chen I, Westwood NJ (2003). Dissecting temporal and spatial control of cytokinesis with a myosin II Inhibitor.. Science.

[65] Fuller BG, Lampson MA, Foley EA, Rosasco-Nitcher S, Le KV (2008). Midzone activation of aurora B in anaphase produces an intracellular phosphorylation gradient.. Nature.

[66] Yoshida S, Kono K, Lowery DM, Bartolini S, Yaffe MB (2006). Polo-like kinase Cdc5 controls the local activation of Rho1 to promote cytokinesis.. Science.

[67] Bahler J, Steever AB, Wheatley S, Wang Y, Pringle JR (1998). Role of polo kinase and Mid1p in determining the site of cell division in fission yeast.. J Cell Biol.

[68] Balasubramanian MK, Bi E, Glotzer M (2004). Comparative analysis of cytokinesis in budding yeast, fission yeast and animal cells.. Curr Biol.

[69] Berdougo E, Nachury MV, Jackson PK, Jallepalli PV (2008). The nucleolar phosphatase Cdc14B is dispensable for chromosome segregation and mitotic exit in human cells.. Cell Cycle.

[70] Skoufias DA, DeBonis S, Saoudi Y, Lebeau L, Crevel I (2006). S-trityl-L-cysteine is a reversible, tight binding inhibitor of the human kinesin Eg5 that specifically blocks mitotic progression.. J Biol Chem.

[71] Vassilev LT, Tovar C, Chen S, Knezevic D, Zhao X (2006). Selective small-molecule inhibitor reveals critical mitotic functions of human CDK1.. Proc Natl Acad Sci U S A.

