# Supplementary figures and images for: Plk1 Self-Organization and Priming Phosphorylation of HsCYK-4 at the Spindle Midzone Regulate the Onset of Division in Human Cells
Source: PLoS Biol. 2009 May 26;7(5):e1000111. doi: 10.1371/journal.pbio.1000111 (PMC2680336; doi:10.1371/journal.pbio.1000111)

Figure S1

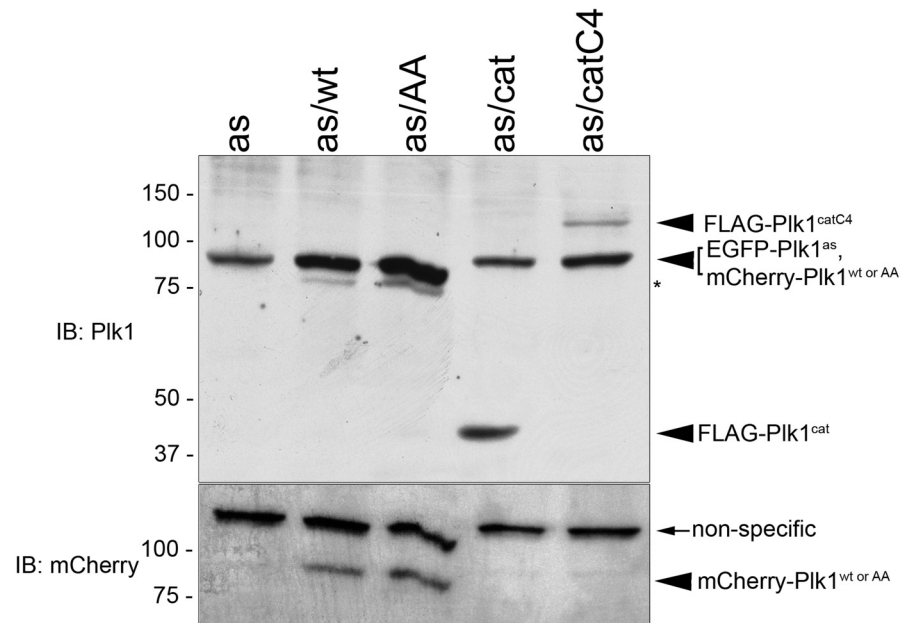

Supplement: Figure S1 — Expression of Plk1 alleles. Whole-cell extracts from cells of the indicated genotypes were resolved by SDS-PAGE and immunoblotted with antibodies specific for Plk1 (top panel) or mCherry (bottom panel). Note that mCherry- and EGFP-fused Plk1 proteins differ by only four amino acids in length and thus comigrate. Asterisk indicates a minor breakdown product of mCherry-Plk1 fusion proteins. A nonspecific band used as a loading control is also indicated. (0.19 MB PDF) [file pbio.1000111.s001.pdf]

Figure S2

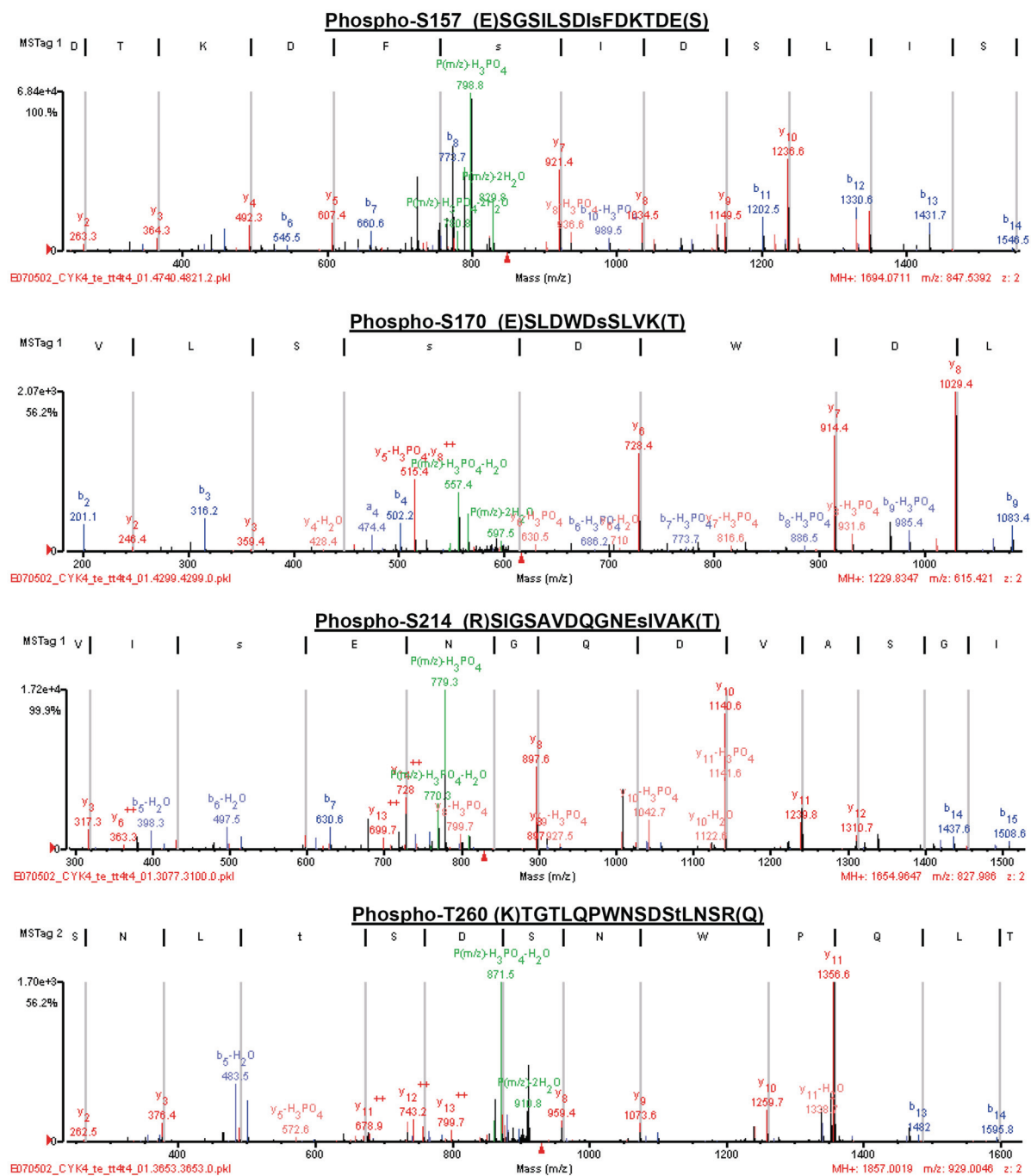

Supplement: Figure S2 — Identification of HsCYK-4 phosphorylation sites by mass spectrometry. A recombinant HsCYK-4N fragment was phosphorylated with purified Plk1 and then processed for tandem mass spectrometry as detailed in the Methods. MS/MS spectra of peptides containing the indicated sites are shown with b-ions in blue and y-ions in red. Ions indicating neutral loss of phosphate from the precursor are shown in green. (0.97 MB PDF) [file pbio.1000111.s002.pdf]

Figure S3

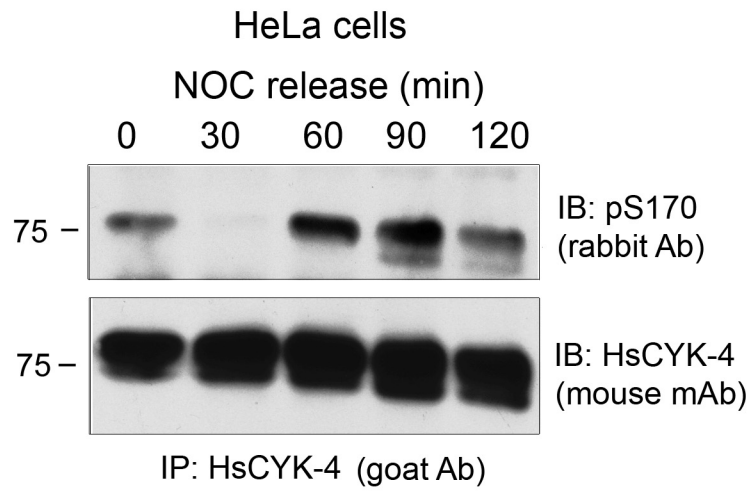

Supplement: Figure S3 — Analysis of pS170 in synchronized HeLa cells. HeLa cells were synchronized by sequential thymidine and nocodazole blocks and released into drug-free medium at time 0. After sampling at various timepoints, whole-cell extracts were prepared and immunoprecipitated with anti-HsCYK-4 antibodies, then immunoblotted to detect either S170 phosphorylation or total HsCYK-4. (0.14 MB PDF) [file pbio.1000111.s003.pdf]

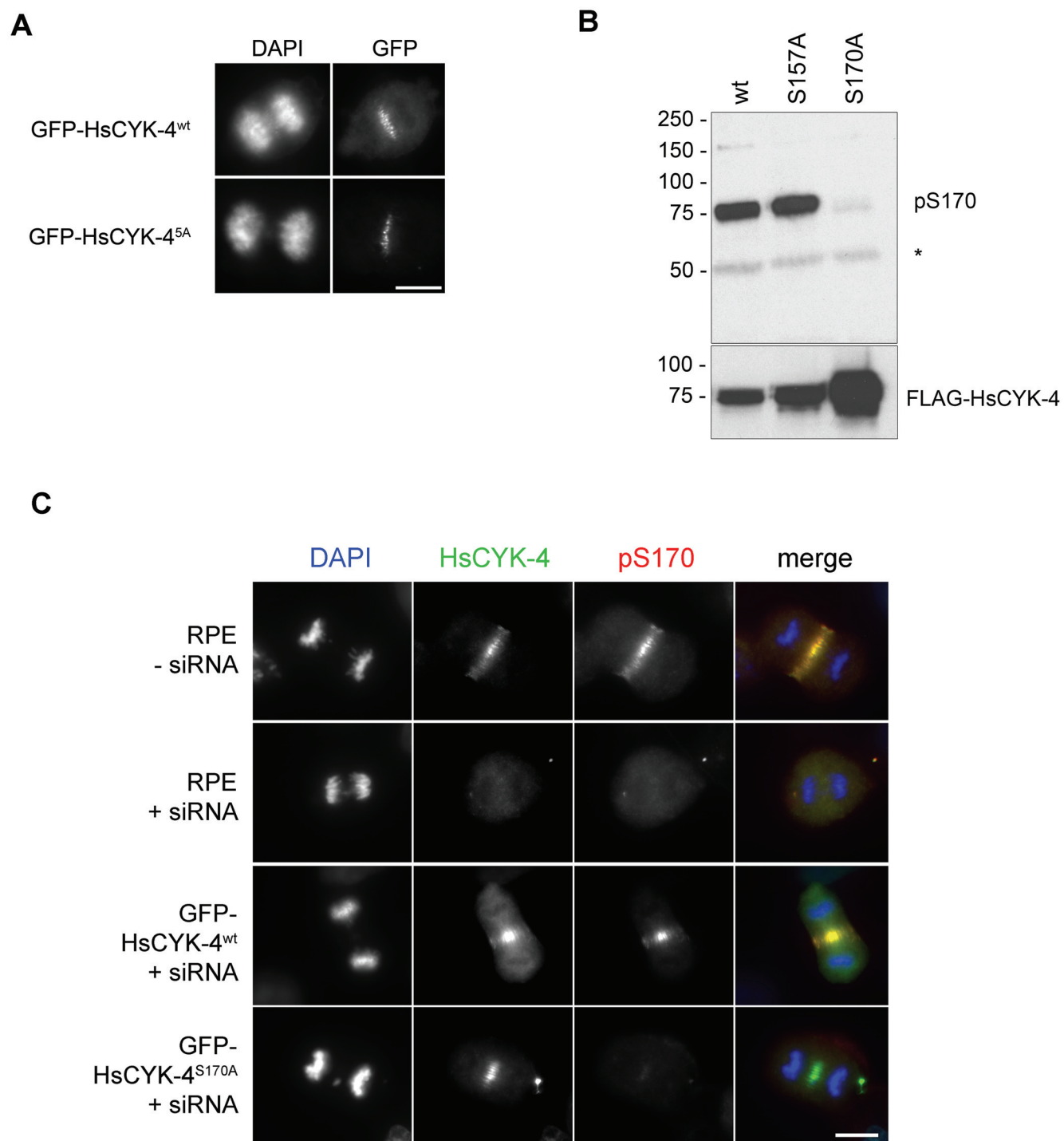

Figure S4

Supplement: Figure S4 — Localization of GFP-HsCYK-4 fusions and validation of the pS170 antibody. (A) RPE cells stably expressing siRNA-resistant GFP-HsCYK-4wt or GFP-HsCYK-45A were stained with a GFP-specific monoclonal antibody and analyzed by fluorescence microscopy. Note comparable incorporation of both GFP-tagged HsCYK-4 alleles into the spindle midzone. Scale bar, 10 µm. (B) HeLa cells were transiently transfected with constructs expressing wild-type, S157A, or S170A versions of FLAG-HsCYK-4 and arrested in mitosis with nocodazole. Extracts were immunoprecipitated with anti-FLAG antibody-Sepharose beads, resolved by SDS-PAGE, and immunoblotted with anti-pS170 antibody (top) or anti-FLAG antibody (bottom). Asterisk denotes crossreaction with immunoglobulin heavy chain. (C) RPE cells were transfected with HsCYK-4 or mock siRNAs, either before (top two rows) or after (bottom two rows) stable transduction with retroviruses expressing siRNA-resistant GFP-HsCYK-4 or GFP-HsCYK-4S170A. Cells were stained with goat antibodies to HsCYK-4 (green) and rabbit anti-pS170 antibodies (red). Note that the S170A transgene restores HsCYK-4 but not pS170. Scale bar, 10 µm. (0.79 MB PDF) [file pbio.1000111.s004.pdf]

Figure S5

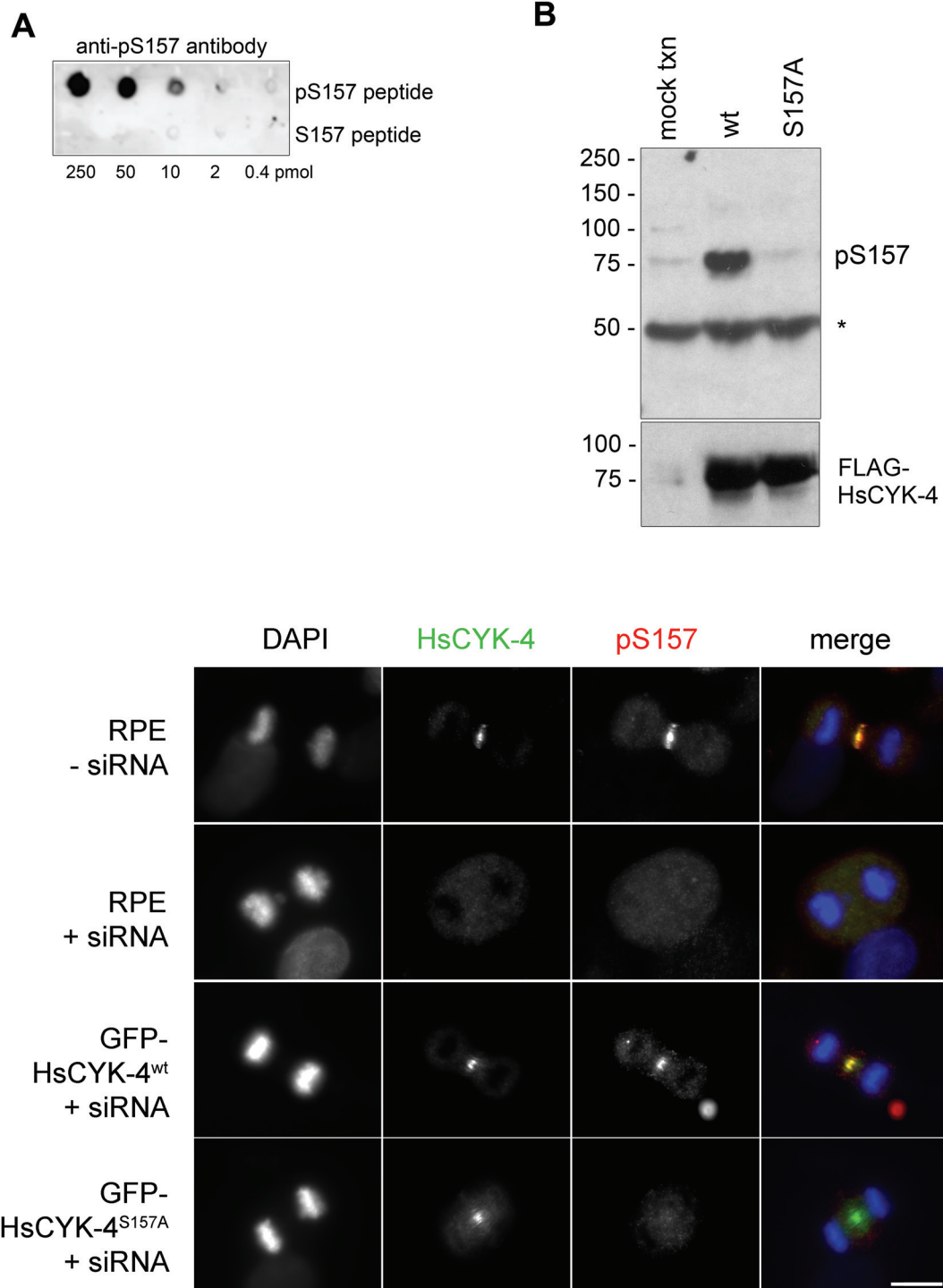

Supplement: Figure S5 — Validation of the pS157 antibody. (A) Decreasing amounts of phosphorylated (pS157) or unphosphorylated (S157) peptides were spotted on PVDF membranes and probed with anti-pS157 antibodies. (B) HeLa cells were transiently transfected with plasmids expressing wild-type or S157A versions of FLAG-HsCYK-4 (or mock-transfected as a negative control) and arrested in mitosis with nocodazole. Extracts were immunoprecipitated with anti-FLAG antibody-Sepharose beads, resolved by SDS-PAGE, and immunoblotted with anti-pS157 antibody (top) or anti-FLAG antibody (bottom). Asterisk denotes crossreaction with immunoglobulin heavy chain. (C) RPE cells were transfected with HsCYK-4 or mock siRNAs, either before (top two rows) or after (bottom two rows) stable transduction with retroviruses expressing siRNA-resistant GFP-HsCYK-4 or GFP-HsCYK-4S157A. Cells were stained with goat antibodies to HsCYK-4 (green) and rabbit anti-pS157 antibodies (red). Note that the S157A transgene restores HsCYK-4 but not pS157. Scale bar, 10 µm. (0.78 MB PDF) [file pbio.1000111.s005.pdf]

Figure S6

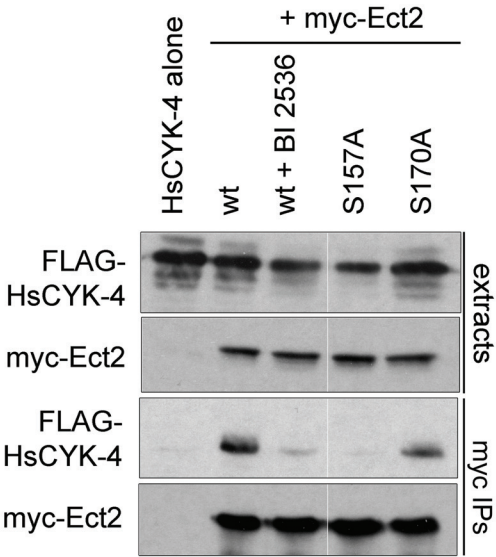

Supplement: Figure S6 — Phosphorylation of serine 157 by Plk1 promotes assembly of the Ect2/HsCYK-4 complex. HeLa cells were transiently transfected with plasmids expressing FLAG epitope-tagged HsCYK-4 (wild-type, S157A, and S170A) and myc epitope-tagged Ect2 (myc-Ect2). Twenty-four hours after transfection, cells were arrested in mitosis for 15 hours with 5 µM S-trityl-L-cysteine [70] (+ 200 nM BI 2536 where indicated), then treated with the Cdk1-specific inhibitor RO-3306 [71] for 20 min to induce mitotic exit. Whole-cell extracts were immunoprecipitated with anti-myc antibodies, resolved by SDS-PAGE, and immunoblotted with anti-FLAG and anti-myc antibodies. (0.34 MB PDF) [file pbio.1000111.s006.pdf]

Figure S7

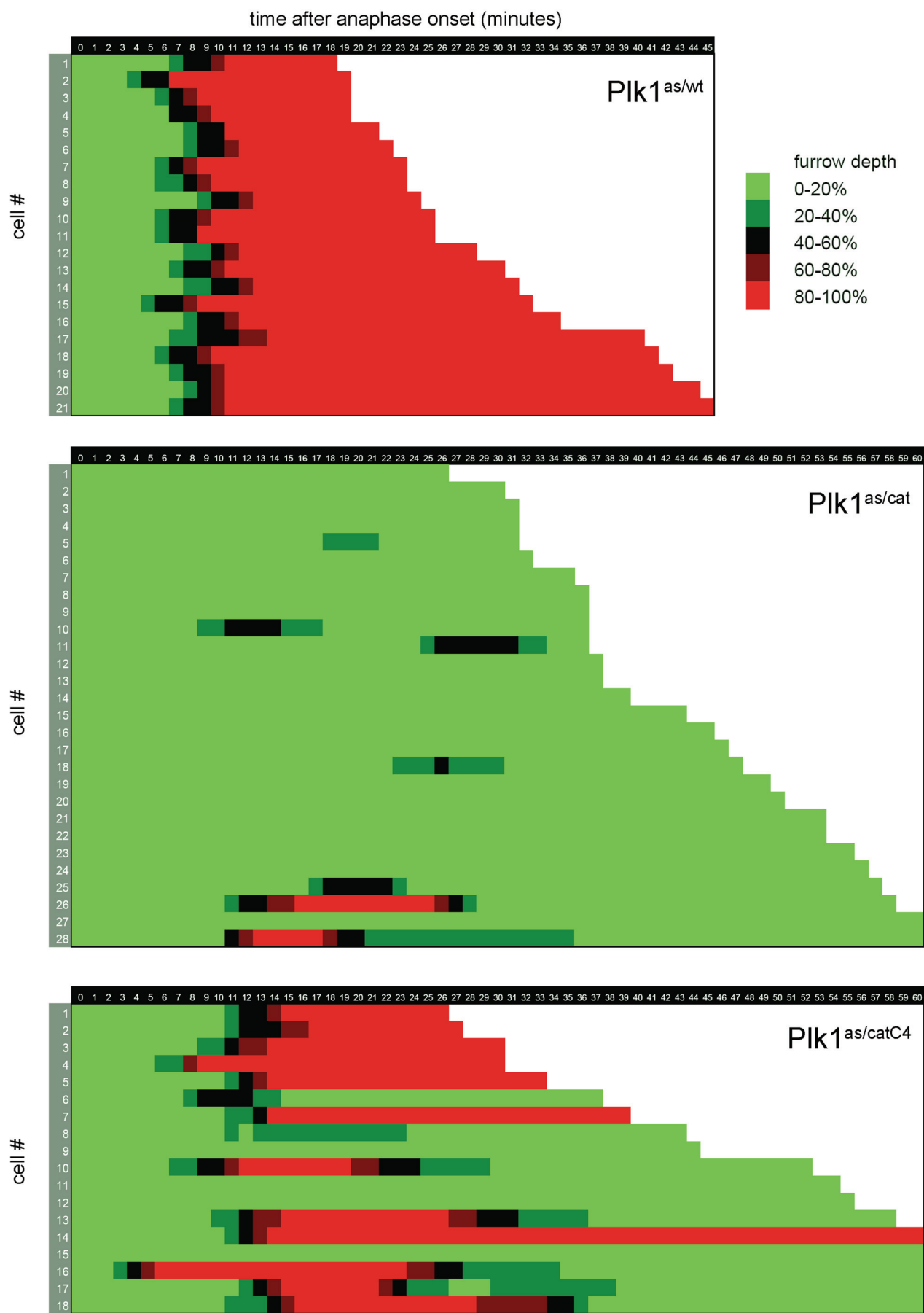

Supplement: Figure S7 — Individual furrow trajectories in Plk1as/wt, Plk1as/cat, and Plk1as/catC4 cells. Cells of each genotype were released from a monastrol block and imaged by phase-contrast videomicroscopy in the presence of 10 µM 3-MB-PP1. The time at which each cell entered anaphase was noted and set as time 0, and the depth of the cleavage furrow was measured at each subsequent frame (1/min) until the cell exited mitosis and adopted a flat morphology. Furrow trajectories are displayed as heat maps to facilitate visual inspection of the dataset. (0.89 MB PDF) [file pbio.1000111.s007.pdf]
